# Assessing the subsistence strategies of the earliest North African inhabitants: evidence from the Early Pleistocene site of Ain Boucherit (Algeria)

**DOI:** 10.1007/s12520-023-01783-8

**Published:** 2023-05-26

**Authors:** Isabel Cáceres, Razika Chelli Cheheb, Jan van der Made, Zoheir Harichane, Kamel Boulaghraief, Mohamed Sahnouni

**Affiliations:** 1 Departament d’Història i Història de l’Art, Universitat Rovira i Virgili, Tarragona, Spain; 2 Institut Català de Paleoecologia Humana i Evolució Social (IPHES-CERCA), Tarragona, Spain; 3 Centre National de Recherches Préhistoriques, Anthropologiques et Historiques (CNRPAH), Algiers, Algeria; 4 Museo Nacional de Ciencias Naturales & Consejo Superior de Investigaciones Científicas (CSIC), Madrid, Spain; 5 Musée Public National du Bardo, Algiers, Algeria; 6 Centro Nacional de Investigación sobre la Evolución Humana (CENIEH), Burgos, Spain; 7 Stone Age Institute & Anthropology Department, Indiana University, Bloomington, IN USA

**Keywords:** Cutmarks, Percussion marks, Subsistence activities, Taphonomy, Oldowan, Early Pleistocene

## Abstract

The archaeological data on the earliest hominin behavioral subsistence activities in North Africa are derived primarily from the Early Pleistocene site of Ain Boucherit (northeastern Algeria). Ain Boucherit consists of two archaeological layers, Ain Boucherit Upper (AB-Up) and Ain Boucherit Lower (AB-Lw), estimated to ~ 1.9 Ma and ~ 2.4 Ma, respectively. Cutmarked and hammerstone percussed bones associated with Oldowan stone tools were found in both layers, with AB-Lw yielding the oldest in North Africa. The faunal assemblages from both deposits are dominated by small-sized bovids and equids. Evidence of cutmarks and percussion marks in both assemblages shows that hominins exploited animal carcasses, involving skinning, evisceration and defleshing activities. The evidence of meat and marrow acquisition is more abundant at AB-Lw with carnivore activity being scarce. However, the AB-Up assemblage shows more carnivore damage and less hominin-induced tool marks. Ain Boucherit evidence, is similar, in type and chronology, to that provided by the Early Pleistocene sites in East Africa (e.g., the Gona sites), where the oldest evidence of stone tools used in faunal exploitation have been discovered. This paper reports on the ability of early North African Oldowans to compete successfully for accessing animal resources with other predators.

## Introduction

The origin of meat consumption by early hominins and its behavioral and dietary implications remains a primary focus of attention in human evolution studies. Currently, it is accepted that, at least by 2.6 million years ago (Ma), human groups used stone tools for early accessing to animal nutrients, at least occasionally (de Heinzelin et al. 1999; Roche et al. 1999; Semaw 2000; Semaw et al. 2003; Domínguez-Rodrigo et al. 2005; Cáceres et al. 2017a). For some researchers, this behavior could have started even earlier, in the Pliocene, as has been raised by the Lomekwi 3 lithic assemblage (Harmand et al. 2015), implying that meat consumption could not be an exclusive behavior to the genus *Homo*.The stratigraphic context of Lomekwi stone tools has been questioned (Domínguez-Rodrigo and Alcalá 2016; Archer et al. 2020), but the exclusivity of the genus *Homo* in the manufacture of tools, or at least in their use, has been questioned after recent discoveries at the Nyayanga site (Kenya), where a *Parantrhopus* molar was found alongside Oldowan tools associated with a butchered hippopotamus skeleton (Plummer et al. 2023). Even so, sites older than 2.0 Ma with stone tools and evidence of hominin access to meat and/or marrow occur. For example, the Ethiopian sites of A.L. 666 (Kimbel et al. 1996), A.L. 894 at Hadar Formation (Hovers 2003) or BD1 at Ledi-Geraru (Braun et al. 2019), and the Kenyan site of Lokalalei 2C (Delagnes and Roche 2005) yielded association of faunal remains with lithic artifacts, although the cut mark scarcity or absence was interpreted as a result of poor preservation of bone surfaces. For instance, the site of Bouri yielded cutmarked bones, but they are not associated with stone tools (de Heinzelin et al. 1999). The absence of stone tools led some archaeologists to question the authenticity of these marks due to the equifinality problems with other agents (Sahle et al. 2017). In North Africa, the oldest evidence has been documented at Ain Boucherit lower and upper archaeological levels and at El Kherba estimated to 2.4, 1.9 Ma, and 1.8 Ma, respectively (Cáceres et al. 2017b; Sahnouni et al. 2013; 2018). The Ain Boucherit butchery evidence is near contemporary with those documented in East Africa.

The sites of this chronology with evidence of a predatory behavior are mainly located in open air context, so the preservation of the bone surfaces is often not optimal for the study of the bone modifications because they were subject to multiples taphonomic processes (weathering, trampling, waterflows, etc.), and there are difficulties to clearly identify the cutmarks and percussion marks (Behrensmeyer 1978; Andrews and Cook 1985, Lyman 1994; Fernández-Jalvo and Andrews 2016; Gifford-Gonzalez 2018; Pineda et al. 2019; Pineda and Saladié 2022).

The accessibility of hominins to animal food resources always depends on multiple factors, whether archaeologically visible or not. Among them are environmental factors such as availability of prey, seasonality, distance to the resources, the size of the hominin group or interaction with other predators, among others (Pobiner 2020). The interaction with carnivores brings us to the discussion on the type of access, i.e., the hunting-scavenging debate (Bunn 1981; Bunn and Kroll 1986; Blumenschine 1988, 1995; Bunn and Ezzo 1993; Domínguez-Rodrigo et al. 2007a, b; Pobiner 2015; Pante et al. 2012; 2015, among others). Thompson et al. (2019) proposed that human predatory behavior began with the use of internal bone nutrients (marrow) rather than with the consumption of meat, whose importance may be overestimated, and, therefore, we would be facing a scavenging strategy. These authors consider that meat consumption and the appearance of knapping tools appear traditionally linked, but that the use of percussive technology must have preceded manufacturing sharp-edged stone tools.

In this regard, a distinction should be made between the sporadic consumption of meat from the regular one. As suggested by Stanford (2012), *Orrorin*, *Sahelanthropus*, or *Ardipithecus*, as well as australopithecines or present-day chimpanzees, may have been able to consume meat opportunistically without necessarily using stone tools. However, the appearance of knapped stone tools meant a significant qualitative difference from opportunistic consumption to regular consumption. At present, whether this regular consumption took place through primary, or secondary, early, or late access is still a matter of debate, even in sites around 2.0 Ma (Blumenschine 1986; Domínguez-Rodrigo 2002; Domínguez-Rodrigo et al. 2007a, b; Pante et al. 2015; Pobiner 2015; Domínguez-Rodrigo and Pickering 2017; Parkinson 2018; Pobiner 2020; Domínguez-Rodrigo et al. 2021, among others).

The evidence that hominins consumed meat regularly is documented at Kanjera South assemblages (KS-1 to KS-3) dated around 2.0 Ma (Ferraro et al. 2013; Oliver et al. 2019). This evidence becomes abundant from 1.9 Ma onward, with a large number of archaeological sites in Africa yielding Oldowan stone tools and associated faunal remains bearing unambiguous cut marks and percussion marks. Sites such as FLK Zinj have traditionally allowed us to reconstruct hominin behavior 1.84 Ma ago, demonstrating that hominins had early access to small-sized prey through hunting and confrontational scavenging to access large animals (Bunn 1981, 2001; Bunn and Kroll 1986; Domínguez-Rodrigo et al. 2007a, b; Domínguez-Rodrigo and Pickering 2017; Bunn and Pickering 2010; Oliver et al. 2019). PTK and DS sites reinforce the interpretations provided by FLK Zinj, demonstrating that Bed I hominins used spaces as referential places where they regularly processed efficiently small- and medium-sized animals obtained by hunting (Cobo-Sánchez 2020; Domínguez-Rodrigo et al. 2021; Organista et al. 2023). For these hominin groups, meat must have played an important role in food sharing, and that they probably developed other complex cooperative behaviors (Parkinson 2018), without ignoring the importance of other types of resources in the hominin diet, such as roots and tubers (USOs) or freshwater resources (Linares-Matás and Clark 2021). The latter is already documented at FwJj20 (Braun et al. 2010) at dates of 1.95 Ma.

We report here the taphonomic analysis of faunal assemblages from Ain Boucherit levels of AB-Lw (2.4 Ma) and AB-Up (1.9 Ma) (Algeria). Both levels provide evidence of animal food resource exploitation and the subsistence strategies conducted by the earliest inhabitants of North Africa.

### Sites background

**Fig. 1:**
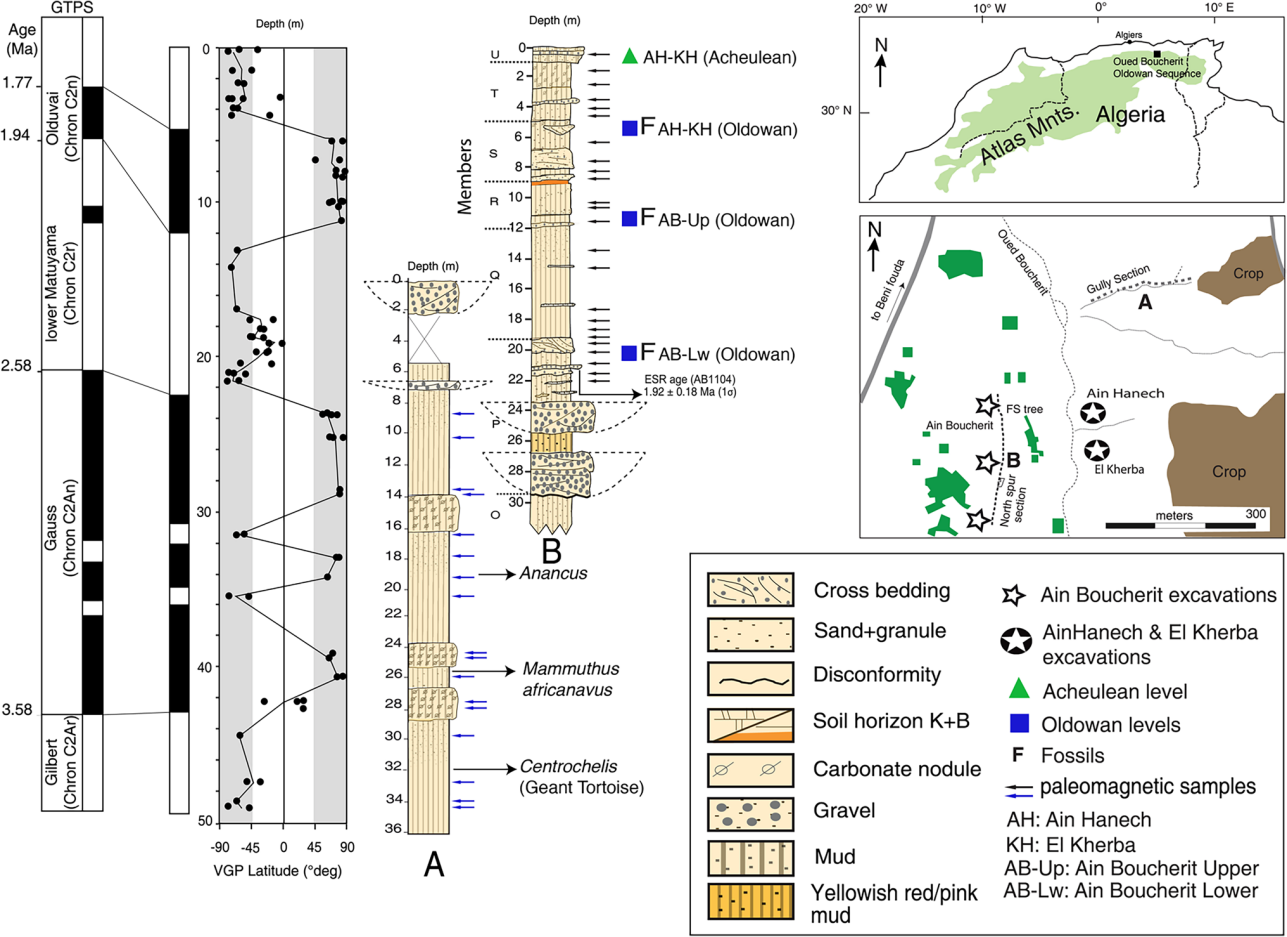
Location of Ain Boucherit-Ain Hanech Oldowan sequence, its stratigraphy and magnetostratigraphy (modified after Sahnouni et al. 2018)

The Ain Boucherit archaeological sites are located on the edge of the eastern Algerian High Plateaus 10 km northeast of the city of El-Eulma in the wilaya (~ province) of Sétif (Fig. 1). They form part of the Ain Boucherit-Ain Hanech research area, which contains a Plio-Pleistocene sequence of archaeo-paleontological deposits, ranging from 3.9 to 1.67 Ma in age (Duval et al. 2021). In addition to Ain Boucherit, the area includes Ain Hanech and El Kherba Oldowan sites (Sahnouni and de Heinzelin 1998; Sahnouni et al. 2002), which are overlain by Acheulean-bearing levels, sealing the sequence. Since 1990s these sites are systematically studied to investigate the tempo and character of the first human occupation in North Africa (Sahnouni in press).

The Ain Boucherit site is situated on the west bank of the intermittent stream Oued Boucherit. It includes two distinct archaeological layers, namely the Ain Boucherit Lower Archaeological level (AB-Lw) (36.2056°N; 5.6527°E) and the Ain Boucherit Upper Archaeological level (AB-Up) (36.2041°N; 5.627°E). AB-Lw refers to the Ain Boucherit fossil bearing stratum that was discovered at the end of the nineteenth century during road works, linking the city of El-Eulma (ex. Saint Arnaud) and the village of Beni Fouda (ex. Silègue). The stratum yielded vertebrate fossils, which were assigned by Pomel (1895, 1897) to the Villafranchian (nowadays chronologically equivalent to the Early Pleistocene) based on the biostratigraphic association of proboscideans (mastodon and *Elephas* sp.) and equids (*Hipparion* sp. and *Equus* sp.). Later, Arambourg (1970, 1979) explored further the stratum, collected more fossils, and confirmed its Villafranchian age. Sahnouni et al. (2002) excavated additional fossils and positioned AB-Lw in the regional stratigraphy and during the 2000s eventually discovered the earliest Oldowan (mode 1) stone tools associated with cutmarked bones in North Africa (Sahnouni et al. 2018). AB-Up is a new archaeo-paleontological level discovered in 2008 in the course of geological work in the vicinity of the reference stratigraphic section. AB-Up is located a few hundred meters south of AB-Lw and 9 m higher in the stratigraphic sequence.

#### Stratigraphy

The Oued Boucherit area is contained in the Mio-Plio-Pleistocene sedimentary basin of Beni Fouda being a sub-basin of the larger Constantine basin with relatively small dimensions (overall about 15 × 15 × 25 km). The Beni Fouda basin is oriented northwest and southeast and is bordered by the Djebel Medjounès to the northwest, by Oulad Sabor to the southwest, and by Djemila anticline to the northeast, while it is opened to the southeast (Vila et al. 1977). On the upper reaches of the Ain Boucherit valley, the sub-basin contains the 100-m-thick informal Oued Laatach Formation and on top of which rests the Ain Hanech Formation of Lower Pleistocene age (Sahnouni and de Heinzelin 1998; Sahnouni et al. 2017).

The Ain Boucherit archaeological levels, along with the relatively younger Ain Hanech and El Kherba deposits, are contained in the 29-m-thick Ain Hanech Formation (Fig. 1). The latter represents a fluvial sequence of braided rivers with pebble and cobble bedload. Its initial 10-m thickness consists of floodplain deposits with very shallow ponds and courses of small rivers that carried sands and small pebbles. The upper part is also fluvial with the formation of a well-developed pedogenic carbonate horizon toward the top of the sequence. The formation comprises, from bottom to top, 6 members: P, Q, R, S, T, and U (Sahnouni et al. 2017, 2018). AB-Lw is encased in the upper part of Mb P, which is 4-m thick and muddy, although with a higher content of clay than the lower part and with sandy intercalations and gravels, suggesting a fluvial system of tributary channels. The lower part consists of river channel facies dominated by gravel and floodplain deposits of pink color (7.5 YR 7/4) or yellow red (5 YR 5/6). AB-Up is encased in Member R that rests immediately on Member Q. The 10- to 15-cm-thick base of Member R consists of a light brown (7.5 YR) sandy mud and well-sorted scatters of gravels of pebble size or small cobbles that diminish in thickness toward the south. This level, along with the overlying 25-cm-thick very pale brown (10 YR 8/2) sandy mud, yielded an Oldowan lithic industry and fossil fauna that is slightly different from that of AB-Lw. The Member R is capped by a light brown (7.5 YR 6/3) mud. These deposits are associated with fluvial gravels and floodplain facies under seasonal flooding conditions. Additionally, the Member T contains the younger Oldowan horizons of Ain Hanech and El Kherba (Sahnouni and de Heinzelin 1998). This 4-m-thick member is light brown (7.5 YR 6/4) or pink (7.5 YR 7/4) in color and is mainly muddy with very hard CaCO3 nodules in the upper 2 m that derive from the overlying Member U as a result of the pedogenic migration of carbonates.

#### The sedimentary context of Ain Boucherit archaeological levels

**Fig. 2:**
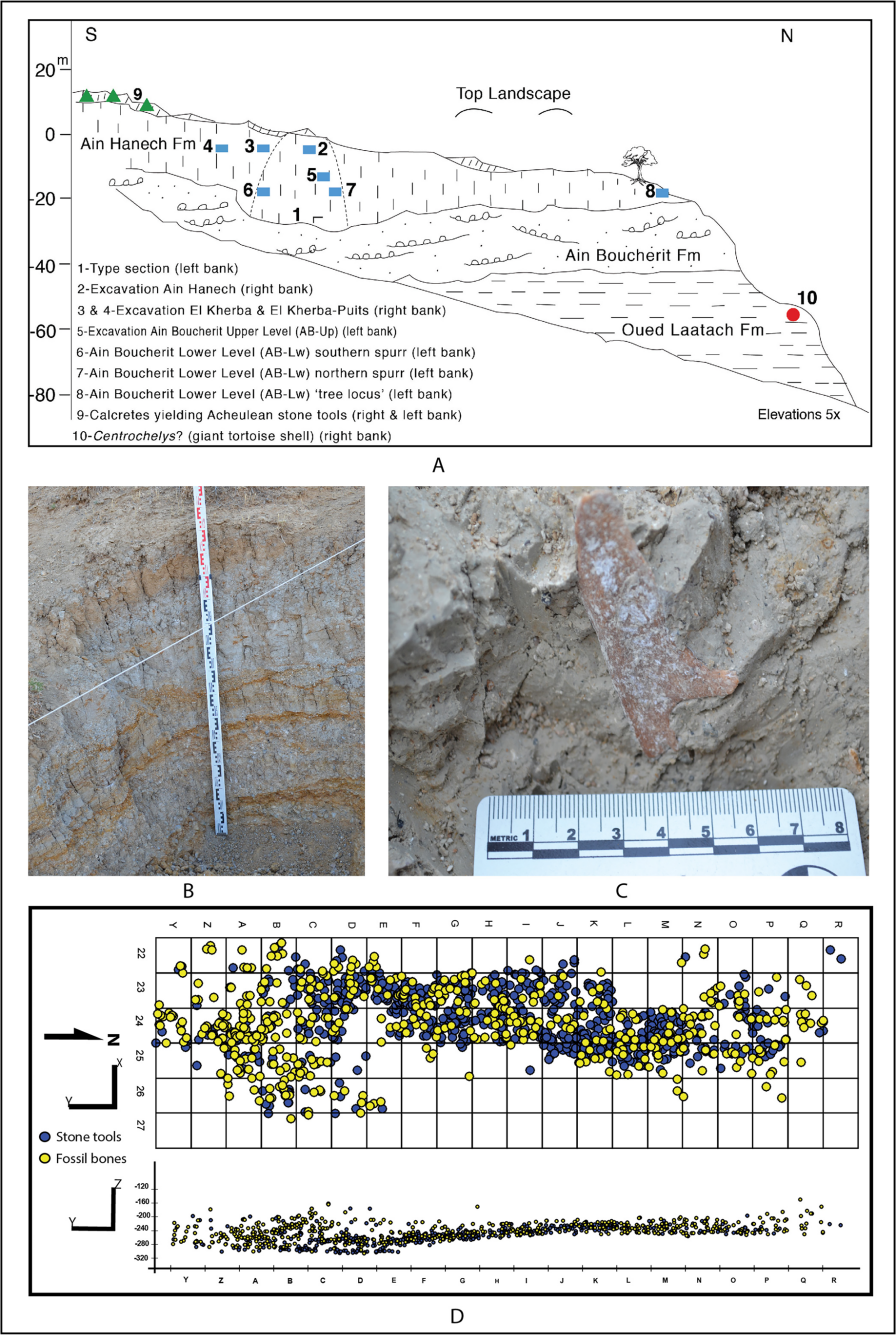
Excavations at AB-Lw and AB-Up, including **A** stratigraphic profile showing the position of Ain Boucherit lower archaeological level (AB-Lw) trench excavations (6, 7, 8) relative to Ain Boucherit upper archaeological level (AB-Up) and to other older and younger paleontological and archaeological deposits (modified after Sahnouni et al. 2018); **B** and **C** trench excavation at AB-Lw in northern spur (7) with a fossil bone being unearthed; and **D** horizontal and vertical projections of fossil bones and associated Oldowan artifacts from the large-scale excavation undertaken at AB-Up

So far, AB-Lw has not been largely excavated due to the difficulty of accessing the archaeological deposit. The AB-Lw archaeological assemblage emanates from limited trench excavations carried out in three locations within the fossil-bearing stratum that extends north–south along the Ain Boucherit hill, including the southern spur, the northern spur, and the “tree locus.” Based on altimetric, stratigraphic, paleontological, and taphonomic evidence, the excavated material in each location belongs to the same fossil-bearing stratum (Sahnouni et al. 2018). The locations of the northern spur (Fig. 2) and southern spur have a great potential for future excavations of AB-Lw. Large-scale horizontal excavations at these two locations are currently not possible due to the steep-walled exposures and ~ 20-m sediment of overburden. The excavations in this deposit are achievable only after entirely excavating the overlying AB-Up deposit and removing at least ~ 8-m thick of sterile sediments separating the two archaeological layers. As indicated in the stratigraphic section above, AB-Lw is encased in the upper part of member P, which is mainly muddy. The sedimentary matrix, sealing fossil animal bones associated with stone tools, is fine-grained made up primarily of silts (83%), fine sands (14%), and clays (2%), suggestive of a floodplain environment (see details in supplementary information in Sahnouni et al. 2018). Silty sediments, which characterize suspended-loaded floodplains, are known to be deposited in a low-velocity flow regime.

The AB-Up archaeological assemblage comes from two layers. The layer at the base is 30-cm thick and consists of a hardened white sandy matrix with cobbles and gravels. The cobbles of various sizes (some over 10 cm in diameter) are unmodified and unaltered. This layer yielded few scattered stone tools and faunal remains. However, the overlying 1.4-m-thick layer produced the bulk of AB-Up archaeological materials. This layer consists of light gray clayey silts within a prismatic soil structure with iron oxide mottles. The sedimentary matrix comprises mainly silt (78%) and clay (18%). Like AB-Lw, the AB-Up stone artifacts and fossil bones were buried primarily in silty sediments suggestive of a floodplain environment setting.

#### Dating

The dating of the Ain Boucherit archaeological levels as well as those of Ain Hanech and El Kherba was undertaken within the entire Oued Boucherit Plio-Pleistocene sedimentary sequence (Duval et al. 2021; Sahnouni et al. 2018). The dating involved a combination of three main dating methods including magnetostratigraphy, Electron Spin Resonance (ESR), and biochronology of large mammals. Stratigraphic columns and the archaeological deposits were sampled for a comprehensive paleomagnetic study, which led to establish a synthetic 50-m-thick magnetostratigraphic sequence with the identification of a succession of intervals of normal and reverse magnetic polarities. The Ain Boucherit archaeological horizons are positioned in an interval of reverse and normal polarity within the Ain Hanech Formation.

An ESR age of 1.92 ± 0.18 million years ago (Ma) (1-σ) was obtained using the multi-center approach on one sample located less than 1 m below AB-Lw level. As a result, AB-Lw in Mb P falls within the lower Matuyama chron, whereas AB-Up correlates to the bottom of the Olduvai subchron. The Ain Hanech and El Kherba deposits are near the top of the Olduvai subchron (1.78 Ma) (Parés et al. 2014; Sahnouni et al. 2018).

Biochronology of large mammals corroborated an early Pleistocene chronology for AB-Lw and AB-Up as well as for Ain Hanech and El Kherba stratigraphically situated in Mb T. For instance, the suid *Kolpochoerus heseloni* (*K. limnetes*) is present at Ain Hanech and El Kherba; its last occurrence dates to around 1.7 Ma (Cooke 2007; White 1995). The proboscidean *Anancus* sp. has been identified in AB-Lw as well as at Ain Hanech, and its most recent occurrences in East, South, and North Africa are dated to around 3.8–3.5 Ma and < 3.1–2.5 Ma (Sahnouni et al. 2018). The presence of these two taxa of biochronological interest in AB-Lw, AB-Up, Ain Hanech, and El Kherba is consistent with their respective correlations with the early Matuyama chron (2.58–1.95 Ma) and the Olduvai subchron (1.95–1.77 Ma) and in agreement with the ESR age. Sediment accumulation rates (SAR) allowed to refine further this chronological interpretation. Consequently, AB-Up and AB-Lw are estimated to be 1.92 Ma and 2.44 Ma, respectively (Parés et al. 2014; Sahnouni et al. 2018).

#### The lithic artifact assemblages

The lithic assemblages excavated from the Ain Boucherit Oldowan sites are made of limestone and flint. These rocks largely occur in the region in several varieties. Their original sources are the rocky hills flanking the Beni Fouda sedimentary basin. They are also found in form of cobbles in several fossil conglomerates throughout the Ain Boucherit area, the densest of which occurs at the base of the Ain Hanech Formation a few meters below AB-Lw. The predominant clast shape available is polyhedral (supplementary information in Sahnouni et al. 2018). These types of limestone and flint cobbles were readily accessible to Ain Boucherit Oldowan knappers for manufacturing their stone tools.

**Fig. 3:**
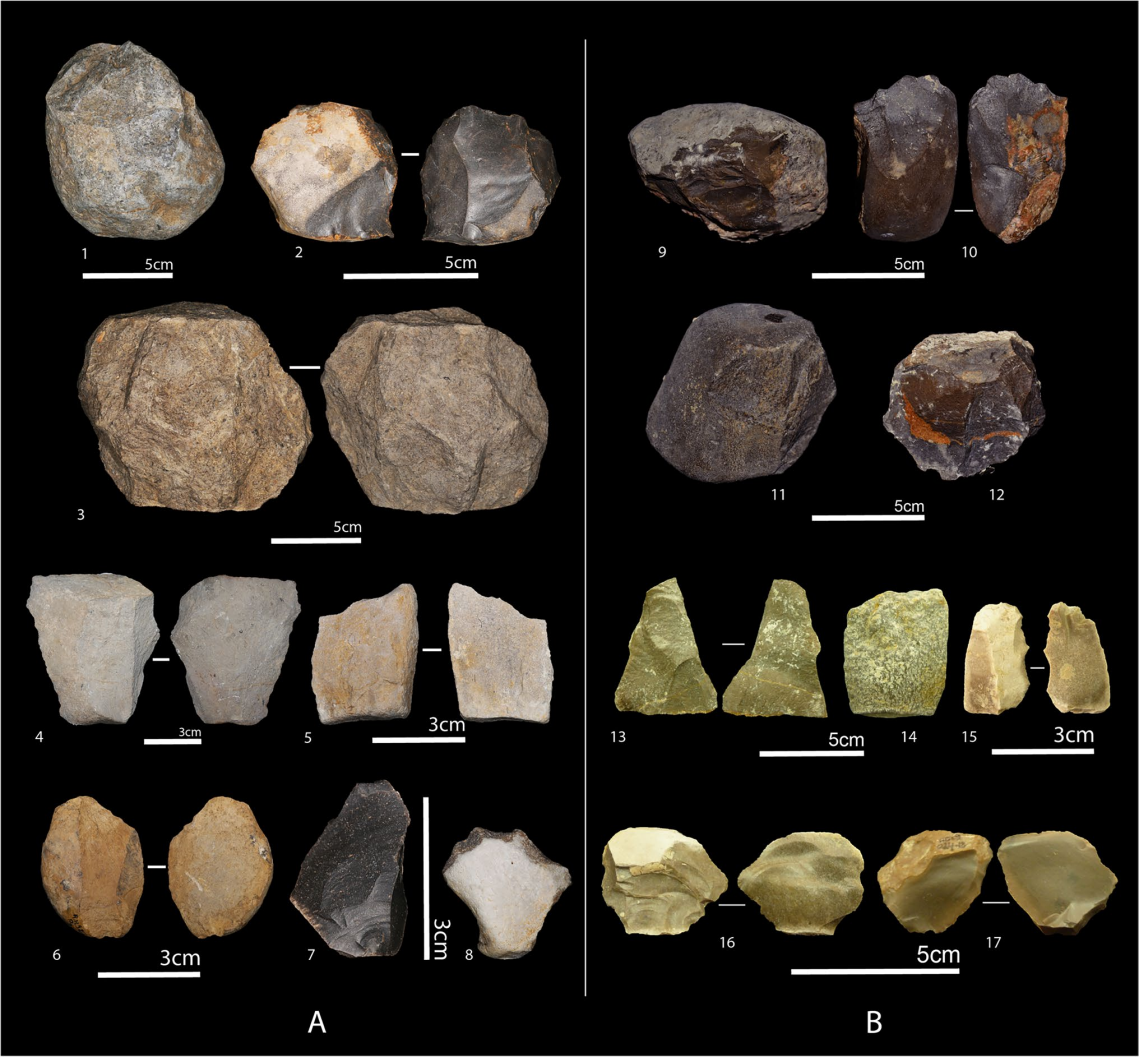
Oldowan stone tools from Ain Boucherit including A (AB-Lw). 1: Unifacial chopper in limestone, 2: core in flint, 3: facetted subspheroid in limestone, 4–6: whole flakes in limestone, 7: whole flake in flint, and 8: notched scraper in flint; B (AB-Up): 9: unifacial chopper in limestone, 10: bifacial chopper in limestone, 11–12: polyhedrons in limestone, 13–14: whole flakes in limestone, 15: denticulate-like in flint, and 16–17: whole flakes in limestone (modified after Sahnouni et al. 2018)

The stone tool assemblages excavated at Ain Boucherit total 17 (AB-Lw) and 236 (AB-Up) specimens (excluding small debitage < 2 cm of maximum dimension) (Table 1, Fig. 3). The AB-Lw is a small assemblage as it derives from limited trench excavations from steep-walled exposures. The AB-Lw stone tools are fresh as they are encased in fine-grained sediments, primarily of silt and clay (Supplementary Information in Sahnouni et al. 2018) and include 7 cores, 9 flakes, and a single retouched piece. The cores (mean dimensions: 85.7 × 67.0 × 55.8 mm, 502.8 g) are primarily polyhedral and irregularly flaked with variable scar counts (2–8 scars). Two cores are flaked using one single striking platform, one onto one face with hinge and step flake terminations, and the other with flaking extending around the platform perimeter. One core is reduced so extensively that it is hard to identify the striking platforms. Half of the associated flakes have cortical dorsal faces and butts. The only retouched piece, measuring 25 × 23 × 10 mm, is a notched scraper made of flint.

**Table 1:** Overall presentation of the Oldowan stone tool assemblages of Ain Boucherit AB-Lw and AB-Up (excluding small débitage < 2 cm of maximum dimension) (modified from Supplementary Information in Sahnouni et al. 2018)

Categories of artifact	Ain Boucherit AB-Lw		Ain Boucherit AB-Up		Total	
	*N*	%	*N*	%	*N*	%
Cores/core forms	7	2.76	121	47.82	128	50.59
Whole flakes	9	3.55	65	25.69	74	29.24
Retouched pieces	1	0.39	3	1.18	4	1.58
Fragments	-	-	47	18.57	47	18.57
Total	17	6.71	236	93.28	253	100

The AB-Up stone assemblage (Table 1) is richer as it is the outcome of a large-scale horizontally excavated area of 81 m^2^ (Fig. 2), yet the density is low, totaling 4.52 artifacts/m^3^. Based on several indications, the bulk of the archaeological material is in primary geological context, within fine-grained sedimentary matrix; high representation of debitage (flakes, fragments, and debris < 2 cm); and absence of (i) preferred orientation, (ii) high inclination, and (iii) noticeable size sorting of artifacts. The lithic assemblage totals 834 (including small debitage < 2 cm) incorporating the following categories: cores and core forms, whole flakes, fragments, and retouched pieces. Most of the cores and core forms were primarily made on limestone than on flint (95.8% vs. 4.13%). They were variably flaked including light (37%), moderate (41%), and heavy (21%), with 50% of the cores still retaining cortex. The cores incorporate unifacial and bifacial choppers (17% and 8%, respectively), polyhedron (23%), subspheroids (2%), spheroids (< 1%), and split cobbles (< 1%). As most cores are on limestone, it follows that most flakes also are on limestone (58.46%) versus on flint (41.53%). The three retouched pieces (mean dimensions: 26.9 × 21.9 × 8.4 mm) are on flint and include two simple scrapers and one notched scraper.

## Material and methods

The taphonomical study involves 906 fossil remains excavated during fieldworks developed until 2019 from Ain Boucherit AB-Lw and AB-Up archaeological levels, totaling 332 (36.6%) and 574 (63.4%) specimens, respectively. The analysis of the faunal remains involved the anatomical and taxonomical identification, indicating the element, position (left or right), portion (proximal/distal end, mid-shaft, proximal/distal metaphysis), and side (anterior, lateral, posterior, and medial) of the faunal specimens. Whenever possible, the age at death of the animal (juvenile, adult, or senile) was recorded, using criteria of tooth eruption and dental wear, and epiphyseal fusion for bones (Stiner 1990). Thus, juvenile category groups the animals with deciduous dentition or non-epiphyseal ends; the adults are individuals with full permanent dentition and fused epiphysis; and senile individuals present advanced tooth wear. For bones without epiphysis, the type of cortical (porous or dense) helps to determine whether the fragment pertains to a juvenile or an adult category, and it is not possible to distinguish the senile category from the adult one. When taxonomical identification was not possible, we used the criteria of mammal size weight categories (Table 2), modified after Bunn et al. (1980) and Bunn (1986). The size categories include (1) very small size refers to class 1A (< 20 kg), (2) small size includes class 1B and 2 (20–100 kg), (3) medium size corresponds to classes 3A and 3B (100–350 kg), (4) large size refers to class 4 (350–1000 kg), and (5) very large size corresponds to classes 5 and 6 (> 1000 kg).

**Table 2:** Classification of the taxa present at the Ain Boucherit site according to weight categories

Size class	Weight range (kg)	Taxa
Very large	> 1000	*Anancus; Mammuthus africanavus; Ceratotherium mauritanicum; Hippopotamus* sp*.; Sivatherium maurusium; "Giraffa" pomeli?*
Large	350–1000	*Pelorovis? Palaethiopicus; Taurotragus gaudryui; Equus numidicus; Equus tabeti*; Equidae indet
Medium	100–350	*Hipparion ambiguum*; *Kolpochoerus* cf. *heseloni*; Suidae indet. *Redunca eulmensis/Parmularius altidens; Connochaetes tournoueri; Parmularius altidens?*
Small	20–100	*Parantidorcas latifrons; Gazella setifensis; Canis primaevus*
Very small	< 20	Antilopinae immature; Vulpes sp.

The abundance of anatomically unidentified bones has led us to also consider bone categories, including (1) *long bones* include specimens with two epiphyses and a medullary cavity (humerus, femur, tibia, radius/ulna, metapodials, fibula, and phalanx), (2) *flat bones* encompass bones with thin cortical and small or no medullary cavity (skull fragments, scapula, rib, coxa, vertebral apophysis), and (3) *articular bones* comprise fragments mainly of spongy tissue (unidentified carpal or tarsal, sesamoid, and unidentified epiphysis fragments). All fossil bones were grouped by skeletal segments: *cranial* (horn, skull, maxilla, mandible, and isolated teeth), *axial* (vertebra, rib, scapula, innominate), *upper limb* (humerus, femur), *intermediate limb* (radius/ulna, tibia), *lower limb* (metapodial, carpal/tarsal, sesamoid, phalanx), and *indeterminate limb* (unidentified long bones).

In order to characterize the Ain Boucherit fossil assemblages, we have calculated the number of identified specimen (NISP), the minimum number of elements (MNE), minimum number of individual (MNI), and the minimum animal units standardized (%MAU). In addition, the correlation between bone mineral density of the %MAU (log) was estimated based on Faith and Behrensmeyer (2006). Bone mineral density data used are provided by Lam and colleagues (1999) for large and medium animals and by Lyman (1994) for small animals. Statistical correlation between %MAU and the utility indexes has been explored. In this sense, the modified general utility index (MGUI) (Binford 1978) and the standardized food utility index (SFUI) (Metcalfe and Jones 1988) have been considered for large-, medium-, and small-sized carcasses.

Regarding bone breakage patterns, we employed the methodology established by Villa and Mahieu (1991) to determine the state of bone (green or dry) during their fragmentation. We analyze the fracture outline (curved, transversal, or longitudinal), the fracture angle (right, oblique, or mixed), and surface texture (smooth or jagged) of all long bones larger than 4 cm. In addition, we recorded the shaft length and shaft circumference of each long bone analyzed.

All bone surfaces were observed macroscopically and microscopically using a stereomicroscope (Optech-LFZ × 45). In some cases, casts with silicone (Provil Novo Heraeus Light) and polyurethane resin (Feropur PR-55/E-55) were made to examine the modifications with a digital microscope Hirox-KH8700 and an environmental scanning electron microscope (FEI-QUANTA 600). Both biostratinomic and fossil-diagenetic modifications were recorded, including modification type, location on bone surface, distribution (isolated, concentrated, scattered, or generalized), and when necessary, the disposition (longitudinal, transversal, oblique, etc.). In addition, whenever possible, we noted the relationships between alterations, with special attention to overlapping modifications.

Cutmarked and intentionally broken bones were analyzed. We examined the type of cutmarks (slicing marks, scraping marks, sawing marks), their location on bone surfaces, their distribution, and their orientation (longitudinal, transversal, oblique) for recognizing the butchery activity process developed (skinning, disarticulation, defleshing, etc.) (Binford 1981; Blumenschine et al. 1996; Nilssen 2000; Lyman 2005; Domínguez-Rodrigo et al. 2009). We analyze the intentional anthropic broken bone, recording the presence and the location of different marks (percussion marks, notches, bone flakes, etc.), whether produced by percussion or bending (Blumenschine and Selvaggio 1988; White 1992; Díez et al. 1999; Fernández-Jalvo et al. 1999; Pickering and Egeland 2006; Alcántara García et al. 2006; Moclán et al. 2019; Vettese et al. 2020).

Carnivore activity was documented by the presence of toothmarks and bone consumption damage. The toothmarks were examined using different methods (Selvaggio 1994; Selvaggio and Wilder 2001; Domínguez-Rodrigo and Piqueras 2003; Delaney-Rivera et al. 2009; and Andrés et al. 2012). The data recorded include the type of toothmarks (pits, scores), their location on skeletal element and bone portion, the type of bone tissue (cortical or cancellous tissue), and the maximum and minimum dimensions of each toothmark in millimeters (mm). Because the tooth mark dimensions themselves are not definitive to identify the carnivore involved, other carnivore damages were also noted such as the presence of furrowing, scooping out, pitting, and digestion evidence (Sutcliffe 1970; Haynes 1980; 1983; Binford 1981; Maguire et al. 1980; Pokines and Kerbis-Peterhans 2007; Esteban-Nadal et al. 2010). In addition, the ratio between preserved epiphyses and diaphysis, as well as the percentage of change obtained for long bones (Marean and Spencer 1991; Marean et al. 1992), was taken into account.

Post-depositional modifications include cracks and exfoliation due to weathering exposure (Behrensmeyer 1978; Lyman 1994), concretions and staining to manganese oxidation (López-González et al. 2006), root-etching (Fernández-Jalvo and Andrews 2016), striae and notches produced by trampling processes (Blasco et al. 2008; Domínguez-Rodrigo et al. 2009), and modification produced by water activity (Cáceres 2002); Fernández-Jalvo and Andrews 2003; Pineda et al. 2019). Related to water activity, some researchers have pointed out the importance of the composition and shape of the fossils as an indicator of fluvial transport of archaeological remains (e.g., Behrensmeyer 1975; Kaufmann et al. 2011; Pante and Blumenschine 2010; Voorhies 1969). Here we have analyzed the possible fluvial transport of the Ain Boucherit faunal assemblages following the criteria of Domínguez-Rodrigo et al. (2014), who considered the bone shapes (flat, tube, or cube) and their composition (dense or trabecular). These researchers compared two different experimental datasets composed by transported and non-transported faunal remains. In addition, Organista et al. (Organista 2017; Organista et al. 2017) compared the former datasets with an undisturbed Masai camp using a correspondence analysis. This test has been performed using the library “cabootcrs” (Ringrose 2019) in R (R Core Team 2023).

## Results

In the AB-Lw faunal assemblage, 332 fossils are assigned to 15 taxonomic groups (Table 3), representing 66.72% of taxonomic identification. The group of bovids is the most represented (NISP = 187–85%), with small-sized bovids (mainly gazelles and *Parantidorcas*) being the most abundant (NISP = 146–66.4%). The total MNI for AB-Lw assemblage is 21, including the jackal *Canis primaevus* and a turtle identified only by its carapace plates. In AB-Lw, the adult individuals constitute the overwhelming majority (90.48%), and the rest includes only one subadult individual (4.76%) of *Parantidorcas latifrons* and one senile (4.76%) of *Parmularius*.

**Table 3:** NISP, MNI, and age of taxa identified at AB-Lw level from Ain Boucherit. Faunal list provided by Van der Made et al. (2021)

Ain Boucherit AB-Lw	NISP	MNI	Juvenile	Adult	Senile
*Anancus osiris*	1	1	-	1	-
*Mammuthus africanavus*	1	1	-	1	-
*Ceratotherium mauritanicum*	1	1	-	1	-
*Hipparion libycum*	2	1	-	1	-
*Equus numidicus*	10	2	-	2	-
*Equus indet*	5	-	-	-	-
*Hippopotamus sp.*	1	1	-	1	-
*Sivatherium maurusium*	1	1	-	1	-
*Pelorovis?*	1	1	-	1	-
*Parmularius? eulmensis/Parmularius altidens*	2	2	-	1	1
*Connochaetes tournoueri*	11	1	-	1	-
*Parantidorcas latifrons*	15	4	1	3	-
*Gazella setifensis?*	18	3	-	3	-
Antilopinae indet	37	-	-	-	-
Bovidae large size	1	-	-	-	-
Bovidae medium size	26	-	-	-	-
Bovidae small size	66	-	-	-	-
Bovidae very small size	10	-	-	-	-
**Total Bovidae indet**	**103**		**-**	**-**	**-**
*Canis primaevus*	2	1	-	1	-
*Mauremys leprosa*	9	1	-	1	-
**Total NISP**	**220**	**21**	**1**	**19**	**1**
Indeterminates	112	-	-	-	-
**Total**	**332**				

Regarding the AB-Up assemblage, 574 remains belonging to 17 taxonomic groups have been analyzed (Table 4). The taxonomic identification rate is lower than that of AB-Lw, reaching 45.8%. The bovids are the most abundant (NISP = 194–73.8%), with the small-sized specimens being the most numerous (NISP = 136–70%). The calculated MNI is 32, and adult animals are the majority (87.88%), followed by juveniles (12.12%). No senile animals are recognized. Carnivore taxa are very minor in both assemblages.

**Table 4:** NISP, MNI, and age of taxa identified at AB-Up level from Ain Boucherit. Faunal list provided by Van der Made et al. (2021)

Ain Boucherit AB-Up	NISP	MNI	Juvenile	Adult	Senile
*Elephas recki ileretensis/Mammuthus meridionalis/ “Elephas moghrebiensis”*	4	1	-	1	-
*Equus numidicus*	14	3	1	2	-
*Equus tibeti*	5	1	-	1	-
Equidae indet	31	-	-	-	-
*Hippopotamus sp.*	2	1	-	1	-
*Kolpochoerus cf. heseloni*	2	2	1	1	-
*Camelus thomasi*	1	1	-	1	-
*"Giraffa" pomeli*	1	1	-	1	-
*Taurotragus gaudryi*	1	1	-	1	-
*Pelorovis*?	1	1	-	1	-
*Gazella cf. setifensis*	23	3	-	3	-
*Parantidorcas latifrons*	28	5	-	5	-
Antilopinae indet	72	6	1	5	-
*Parmularius altidens*?	10	2	-	2	-
*Connochaetes tournoueri*	2	2	1	1	-
*Alcelaphinae indet*	4	-	-	-	-
Bovidae large size	1	-	-	-	-
Bovidae medium size	39	-	-	-	-
Bovidae small size	13	-	-	-	-
**Total Bovidae indet**	**53**	**-**	**-**	**-**	**-**
*Ursus* sp.?	1	1	-	1	-
*Canis primaevus*	6	1	-	1	-
Felidae large size	2		-	-	-
**Total NISP**	**263**	**32**	**4**	**28**	**-**
Indeterminates	311		-	-	-
**Total**	**574**				

Skeletal representation by weight categories (Table 5) is similar for all categories, based on the NISP and MNE for each level, although large-sized animals are more abundant at the AB-Up level and with a better skeletal representation. At both levels, the most complete skeletal profiles correspond to small and medium sizes, while the large and very large sizes show very biased representations, since represented mainly by isolated dental pieces or axial fragments (1 rib from AB-Lw and 2 vertebra fragments from AB-Up).

**Table 5:** NISP and MNE in brackets by size category at AB-Lw and AB-Up faunal assemblages. Remains belonged to carnivores and tortoise are not included

	Very large		Large		Medium		Small		Very small		Indeterminate		Total	
	AB-LW	AB-UP	AB-LW	AB-UP	AB-LW	AB-UP	AB-LW	AB-UP	AB-LW	AB-UP	AB-LW	AB-UP	AB-LW	AB-UP
	MNI = 5	MNI = 3	MNI = 3	MNI = 6	MNI = 4	MNI = 4	MNI = 5	MNI = 15	MNI = 2	MNI = 2			MNI = 19	MNI = 30
Horn	-	-	-	-	3 (1)	3 (1)	18 (8)	34 (15)	2 (1)	2 (2)	-	-	23 (10)	39 (18)
Skull	-	-	-	2 (1)	-	5 (4)	7 (3)	9 (3)	1 (1)	1 (1)	3 (-)	2 (-)	11 (4)	19 (9)
Maxilla		-		-		-		2 (2)		-		-		2 (2)
Mandible	-	-	-	2 (1)	1 (1)	3 (2)	5 (4)	7 (6)	1 (1)	-	-	-	7 (6)	12 (9)
Isolated tooth	5 (-)	6 (-)	7 (-)	24 (-)	11 (-)	32 (-)	17 (-)	24 (-)	2 (-)	3 (-)	1 (-)	2 (-)	43 (-)	91 (-)
Vertebra	-	2 (2)	2 (2)	-	4 (4)	2 (2)	4 (4)	1 (1)	7 (7)	-	1 (-)	-	18 (17)	5 (5)
Rib	1 (1)	3 (2)	4 (3)	5 (3)	2 (1)	13 (5)	7 (2)	2 (1)	1 (1)	1 (1)	-	-	15 (8)	24 (12)
Scapula	-	1 (1)	-	1 (1)	2 (1)	5 (5)	4 (3)	1 (1)	1 (1)	-	-	-	7 (6)	8 (8)
Humerus	-	-	-	1 (1)	2 (2)	3 (2)	8 (3)	3 (3)	1 (1)	1 (1)	-	-	11 (6)	8 (7)
Radius	-	-	-	3 (3)	3 (2)	1 (1)	12 (7)	3 (2)	3 (3)	-	-	-	17 (12)	7 (6)
Ulna	-	-	-	-	-	-	2 (1)	3 (3)	1 (1)	-	-	-	3 (3)	3 (3)
Carpal	-	-	-	3 (3)	-	1 (1)	1 (1)	1 (1)	-	-	-	-	1 (1)	5 (5)
Metacarpal	-	-	1 (1)	4 (4)	3 (3)	3 (3)	9 (7)	5 (3)	2 (2)	-	-	-	15 (13)	12 (10)
Innominate	-	-	-	1 (1)	-	1 (1)	3 (3)	2 (2)	1 (1)	1 (1)	2 (-)	-	6 (4)	5 (5)
Femur	-	-	-	2 (1)	1 (1)	3 (2)	4 (4)	4 (2)	6 (2)	-	-	-	11 (7)	9 (5)
Tibia	-	-	3 (3)	10 (9)	3 (1)	5 (4)	8 (5)	7 (5)	-	1 (1)	-	-	14 (9)	23 (19)
Tarsal	-	-	4 (4)	2 (2)	4 (4)	1 (1)	7 (7)	3 (3)	1 (1)	-	-	-	16 (16)	6 (6)
Metatarsal	-	-	-	1 (1)	2 (2)	4 (3)	12 (8)	5 (4)	3 (2)	-	-	-	17 (12)	10 (8)
Metapodial	-	-	2 (2)	1 (1)	2 (1)	5 (-)	4 (1)	10 (1)		-	-	-	8 (4)	16 (2)
Phalanx	-	-	-	1 (1)	5 (5)	-	12 (12)	16 (16)	-	-	-	-	17 (17)	17 (17)
Sesamoid	-	-	-	-	-	-	2 (2)	1 (1)	-	-	-	-	2 (2)	1 (1)
Long bone	-	-	2 (-)	15 (-)	8 (-)	79 (-)	5 (-)	52 (-)	-	8 (-)	4 (-)	6 (-)	19 (-)	160 (-)
Flat bone		1 (-)		-		12 (-)		4 (-)		-		11 (-)		27 (-)
Indeterminate	2 (-)	3 (-)	-	7 (-)	-	5 (-)	-	3 (-)	-	-	37 (-)	37 (-)	39 (-)	55 (-)
Total	8 (1)	16 (5)	25 (15)	85 (33)	56 (29)	186 (37)	151 (85)	202 (75)	33 (25)	18 (7)	48 (-)	58 (-)	321 (155)	565 (157)
Total%	2.49 (0.65)	2.80 (3.18)	7.79 (9.68)	14.89 (21.02)	17.45 (18.71)	32.57 (23.57)	47.04 (54.84)	35.38 (47.477)	10.28 (16.13)	3.15 (4.46)	14.95 (-)	10.16 (-)		

**Fig. 4:**
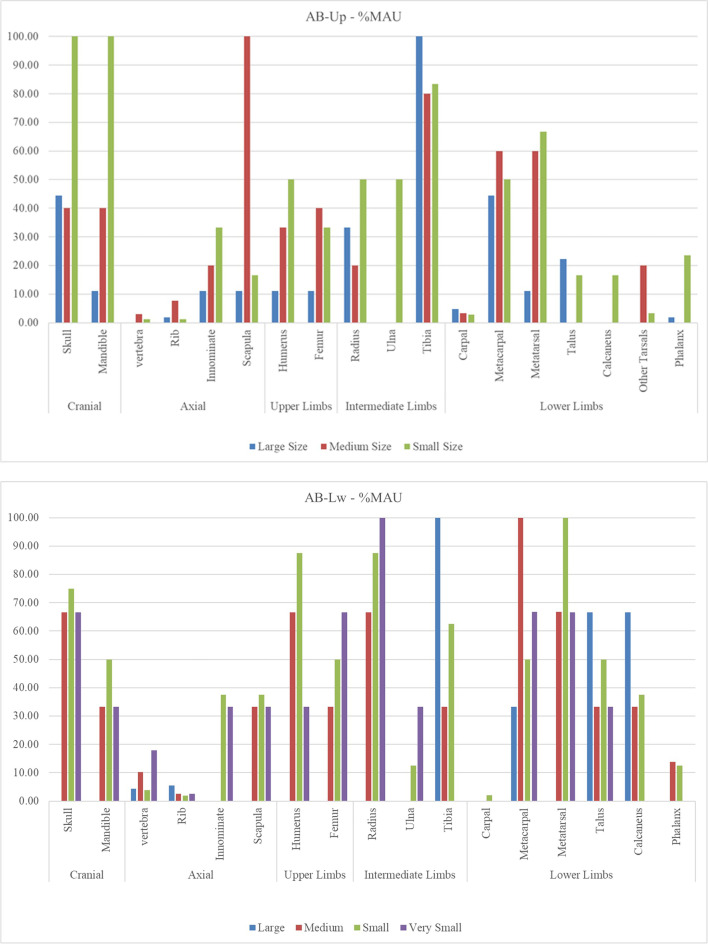
%MAU according to weight category and skeletal segment for AB-Up (top) and AB-Lw (bottom). In AB-Up, very small size has not been included due to the low number of elements provided

The %MAU obtained for the AB-Lw level (Fig. 4) indicates that for medium- and small-sized animals, the lower limbs are the best represented, followed by skulls, while the axial is underrepresented. This representation is different for the large-sized animals, which has not provided cranial remains and the appendicular is only represented by tibiae and lower limbs. At the AB-Up assemblage, the skeletal representation is similar in all size categories, with predominance of heads and limbs.

The correlation between %MAU and bone mineral density (Table 6) indicates, for both assemblages, that there is no differential preservation of remains related to bone density for any of the animal weight categories. However, only the small animals of both assemblages and the medium-sized animals of AB-Up have provided statistically significant values.

**Table 6:** Correlation values of %MAU and bone mineral density of animals from AB-Lw and AB-Up

	Large size		Medium size		Small size	
	*r*	*p*-value	*r*	*p*-value	*r*	*p*-value
AB-Lw	0.1964	0.0605	0.2336	0.0219	0.4489	0.000067
AB-Up	0.1476	0.1578	0.3220	0.0013	0.457	0.0010

In the AB-Lw level, the results of the correlation between %MAU and MGUI for medium- and small-sized animals indicate a statistically significant correlation with *p* = 0.0005 and *r* = 0.75; *p* = 0.0008 and *r* = 0.73, respectively. This correlation in the medium-sized elements from AB-Up shows a statistically non-significant correlation *p* = 0.039 and *r* = 0.53. However, in the case of small elements, we observe a more or less significant correlation *p* = 0.006 and *r* = 0.64. Considering the value of Evenness (*e*), according to the method of Faith and Gordon (2007), and taking into account that the MNE is less than 50 elements for all categories, the nutritional strategy at AB-Lw may correspond to a strategy close to the complete transport of both, medium- and small-sized animals (Table 7). In AB-Up, the value of Evenness (e) shows a statistically positive correlation for medium-sized and small elements. Nevertheless, the Spearman’s (rho) application showed that small animals were likely transported more or less complete while medium- and large-sized animals respond to an *Unconstrained* transport strategy.

**Table 7:** The SFUI and MNE values of high survival elements and the *Evenness (e)* index results, Spearman’s *ratio* values to AB-Lw and AB-Up assemblages

(S)FUI	AB-Lw			AB-Up		
	L	M	S	L	M	S
*(e) Evenness*	0.4231395	0.9414210	0.9525259	− 0.5757576	0.94931969	0.9582258
*(rho) Spearman’s*	0.8734648	− 0.2363636	0.2969697	− 0.7272727	0.03636364	0.2484848

### Bone breakage

At Ain Boucherit, the percentage of fragmented bones is very high, reaching 86.4% at AB-Lw and 92.82% at AB-Up. The complete bones recovered correspond mostly to phalanges and articular bones (carpals, tarsals, and sesamoids). Horns also appear fragmented in 93.10% for AB-Lw and 97.8% at AB-Up. In both levels, abundant isolated teeth occur, representing 18.96% of the remains at AB-Lw and 16.72% at AB-Up, which points out, at the same time, the low integrity of the maxillae and mandibles.

**Fig. 5:**
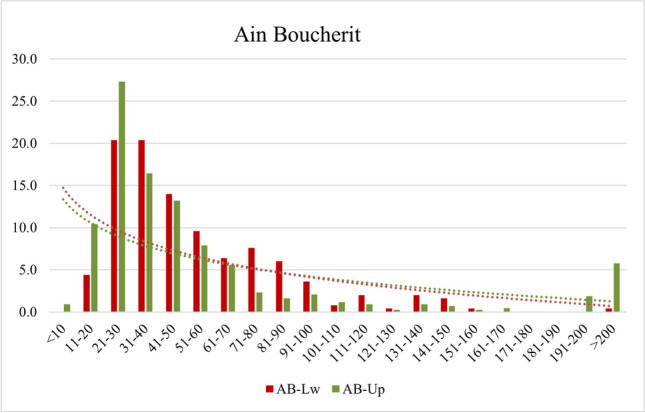
Length of the fossils recovered at each faunal assemblage grouped by ranges of 10 mm

The degree of fragmentation of these fossil associations is also reflected if we consider the dimensions of the recovered remains. Thus, 59.2% of the remains at AB-Lw and 67.3% at AB-Up do not exceed 5 cm in length (Fig. 5), which are high percentages considering the presence of large and very large taxa.

**Fig. 6:**
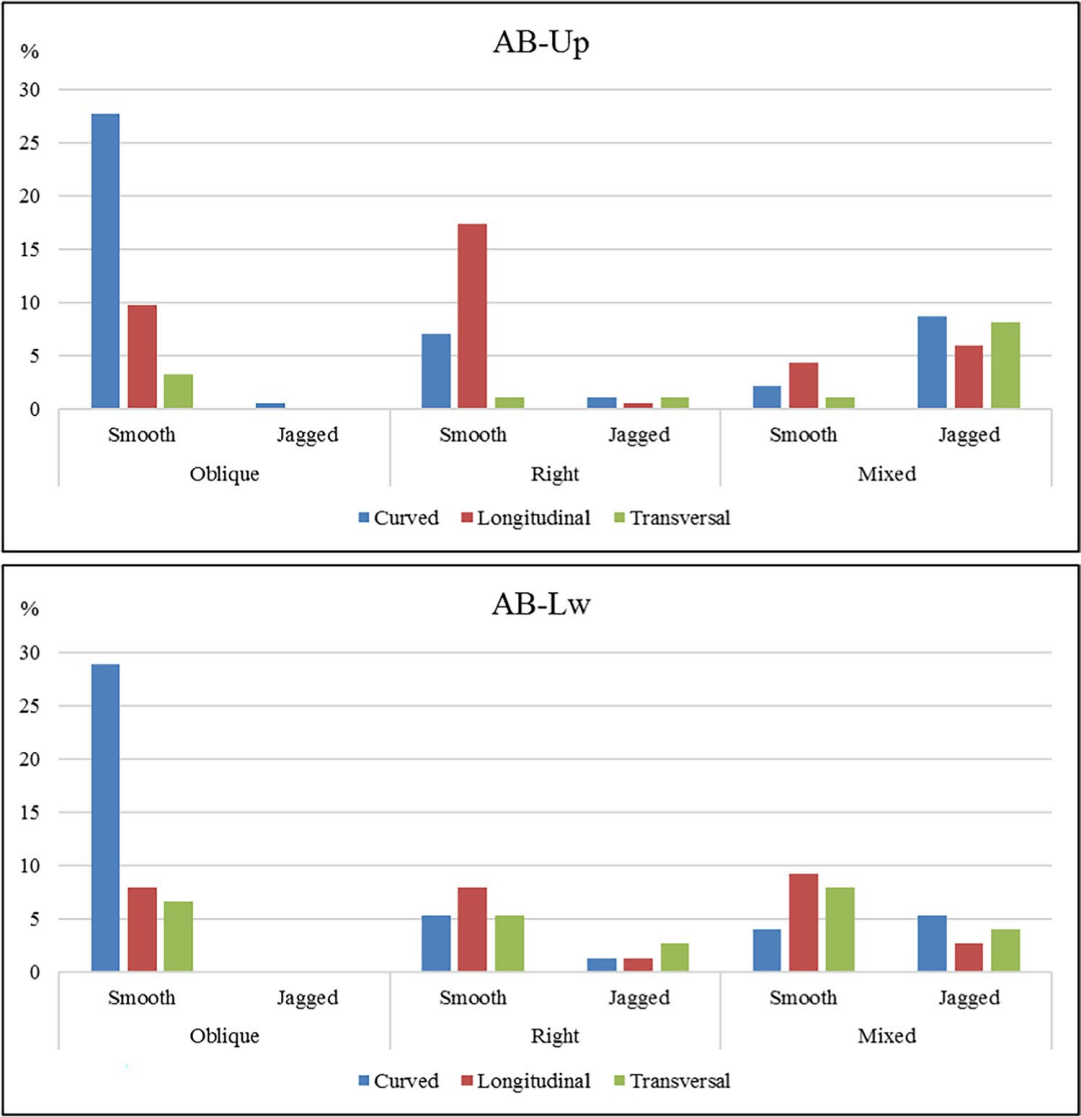
Delineation, angle, and surface type of fractures identified on long bones from AB-Up (top) and AB-Lw (bottom) following Villa and Mahieu (1991)

At AB-Lw, 26 fractured long bones with a total of 76 fracture surfaces have been analyzed (Fig. 6). The dominant delineation is curved (44.74%), followed by longitudinal (28.95%) and transverse (26.32%). Oblique angles are the most abundant (43.42%), followed by mixed angles (32.89%) and right angles (26.32%). As for the surfaces, smooth surfaces are predominant (82.89%). Regarding the diaphysis length, there is a balance between fragments showing < ¼ of the original length and those showing between ½ and ¾, representing 38.5%. On the other hand, 61.54% of the analyzed long bones preserve complete circumferences.

At AB-Up (Fig. 6), 184 fracture surfaces belonging to 48 long bones were recognized. A total of 47.28% of the fractured long bones show curved delineation, followed by longitudinal delineation (38.04%) and by transverse delineation (14.67%). The angles are mostly oblique (40.76%), followed by mixed angles (30.43%) and right angles (28.26%). Smooth surfaces (73.91%) predominate the jagged fractures (26.09%), although the values are slightly minor than in the AB-Lw level. Another difference between both assemblages is that, in AB-Up, < ¼ shaft length predominates (75%) and the preserved shaft circumferences are mainly < ½ (56.25%), suggesting a higher degree of fragmentation at AB-Up.

Bone breakage analysis suggests that, at both assemblages, the bone breakage occurred when the bones were in a green state. Thus, curved or longitudinal fractures with oblique angles reach 36.84% at AB-Lw and 37.50% for AB-Up, suggesting that the bones broke predominantly when fresh, while, in 19.74% (AB-Lw) and 11.41% (AB-Up), the state of the bones was dry (transverse delineation with right or mixed angles). This result suggests the involvement of several taphonomic agents responsible for bone breakage.

Considering the longitudinal/oblique ratio for bone fracture planes and comparing them with data provided by some experimental assemblages (Table 8), we observe that at Ain Boucherit, the small and medium-sized animals present values below 1, related to anthropic hammerstone-percussed assemblages. Only the large-sized animals of AB-Up show values above 1, referring to carnivore bone breakage datasets. At AB-Lw, values provided by medium-sized carcasses, closer to 1, would indicate that carnivores would were also involved.

**Table 8:** Ratio longitudinal versus oblique fracture planes from large (LS), medium (MS), and small-sized animals from Ain Boucherit levels and other fossil and experimental assemblages. Comparative dataset provided by ^a^ = Moclán et al. 2019; ^b^ = Alcántara-García et al. 2006; ^c^ = Coil et al. 2017

	Longitudinal	Oblique	Ratio
AB-Lw MS	11	12	0.92
AB-Lw SS	5	17	0.29
AB-Up LS	10	8	1.25
AB-Up MS	34	48	0.71
AB-Up SS	16	24	0.67
Percussion MS^a^	297	549	0.54
Hyenas MS^a^	91	87	1.05
Wolves MS^a^	287	273	1.05
Percussion LS^b^	50	86	0.58
Percussion SS^b^	37	54	0.69
Carnivore LS^b^	21	21	1.00
Carnivore SS^b^	16	14	1.14
Percussion MS^c^	265	441	0.60
Carnivores MS^c^	31	76	0.41

### Bone surface modifications

**Fig. 7:**
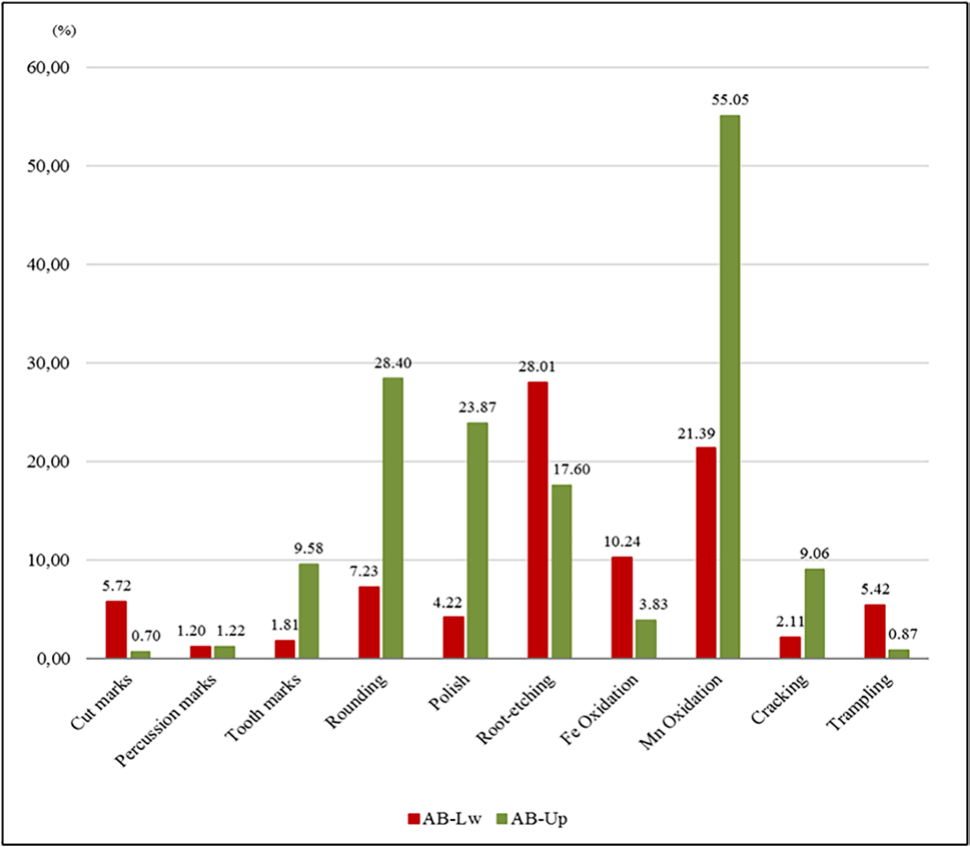
Bone surface modifications identified on fossils from AB-Lw and AB-Up faunal assemblages

Different types of bone surface modifications have been recorded on Ain Boucherit fossils, including modifications related to anthropogenic and carnivore activities, as well as to post-depositional processes (Fig. 7).

The post-depositional modifications are the most abundant at both assemblages (Fig. 7). Pigmentations produced by manganese (Mn) and iron (Fe) oxides, grooves caused by plant roots, and the rounded and polished surfaces related to water activity are noteworthy. These modifications are manifested differently at each faunal assemblage, but, in general, they are more abundant at the AB-Up level. In AB-Lw, root marks (28.01%) are the most frequent alteration, followed by Mn (21.39%) and Fe (10.24%) oxidations. Other modifications do not exceed, in any case, the frequency of 8%. At the AB-Up level, Mn oxidations reach 55.05%, followed by water-abraded surfaces: rounding (28.40%) and polishing (23.87%). The abrasion appears in moderate grades and mainly affects partially the surface of the specimens. Root activity is lower (17.60%) at AB-Up. Cracking is more abundant at the AB-Up level (9.06%), while trampling does not reach 1%. Yet, this is reversed at the AB-Lw level, where trampling is higher (5.42%) and weathering is lower (2.11%). The relationship between the post-depositional modifications in each of the assemblages has resulted in better preservation of the surfaces at AB-Lw level than at AB-Up.

**Fig. 8:**
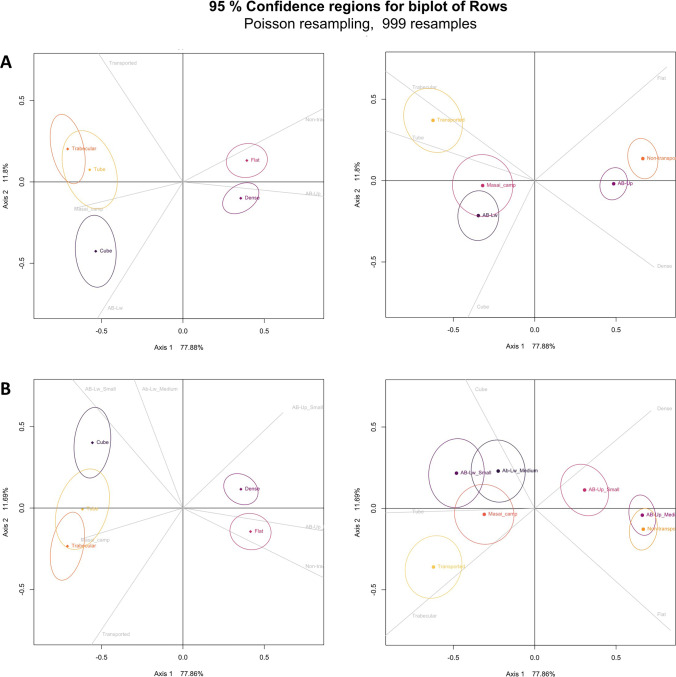
Bootstrapped correspondence analysis with the composition of bones (dense, trabecular) and their shape (cube, flat, tube) at AB-Lw and AB-Up faunal assemblages compared with different water transport models (Domínguez-Rodrigo et al. 2014; Organista 2017; Organista et al. 2017). Ellipses indicate the 95% CIs. **A** Biplots with general data for each level and **B** according to medium- and small-sized carcass. The distribution of the bone types (left) and distribution of the faunal assemblages providing these bone data (right)

Regarding the percentages of water abrasion identified, it was necessary to consider the role of fluvial transport in the formation of the Ain Boucherit archeological levels. Thus, the analysis of specimen shape and composition (Domínguez-Rodrigo et al. 2014; 2019) places the AB-Lw closer to Masai camp and AB-Up assemblages next to nontransported comparative assemblages (Fig. 8). The distribution of each level could be explained by the higher representation of cube remains and specimens with epiphysis at AB-Lw than at AB-Up. The correlation between bone shape and composition suggests that both faunal assemblages show no significant hydraulic disturbance. Consequently, they are suitable assemblages for elucidating the strategies developed by hominins and carnivores.

#### Hominin activity

**Fig. 9:**
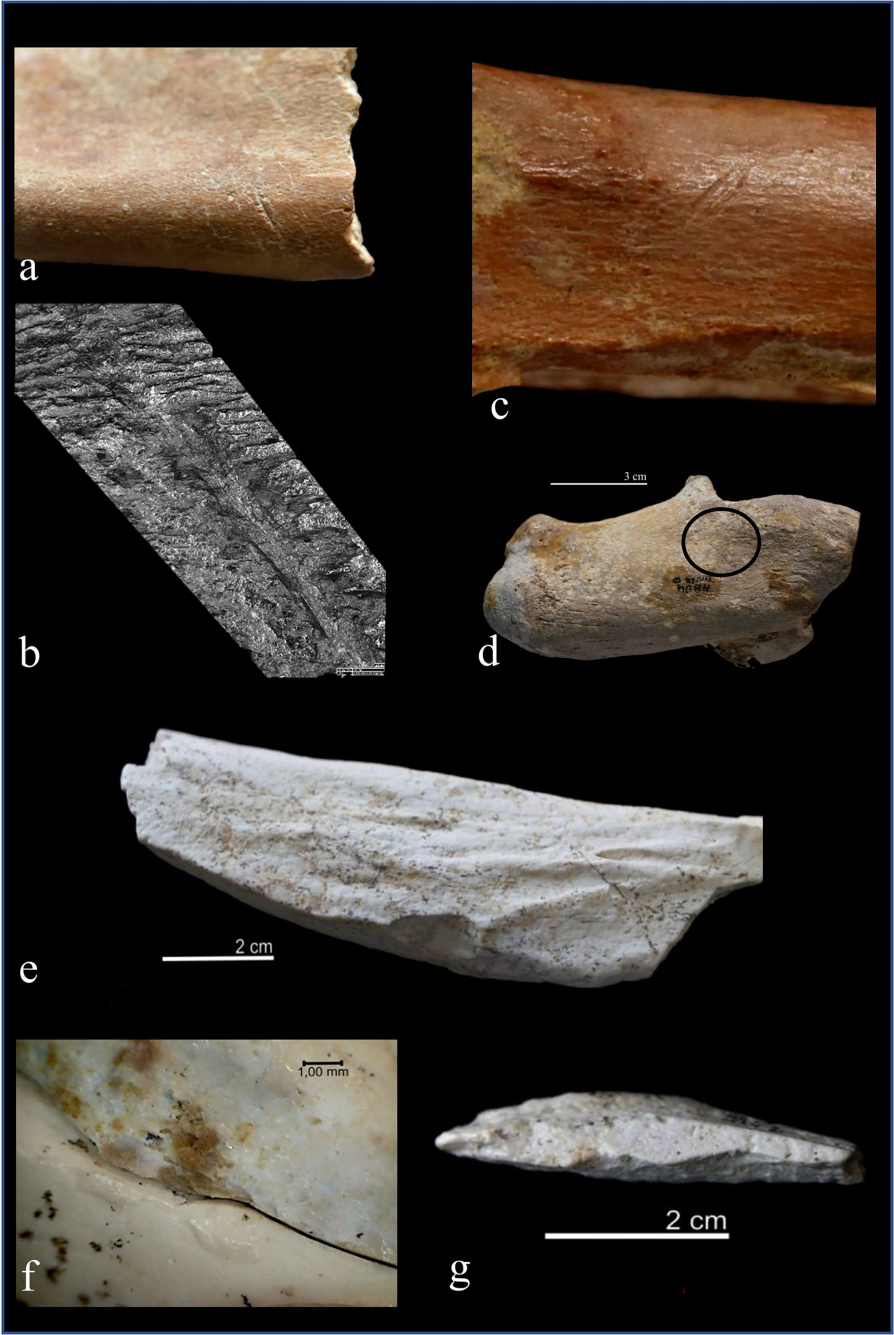
**a** Cutmarks on a small bovid mandible from AB-Lw and **b** detail obtained with a digital microscope Hirox KH 8700. Note de V-shape section, the internal microstriation, and Hertzian cones. **c** Cutmarks on a small bovid radius from AB-Lw; **d** cutmark on a Equidae calcaneum from AB-Lw; **e** Equidae tibia shaft from AB-Up with cortical scars; **f** refit between two long bones from AB-Up, note the impact related to bone breakage; **g** bone flake with percussion platform from AB-Up.

Hominin activity consists of cut marks and percussion marks related to meat and marrow exploitation. They occur unevenly in the two archaeological levels of Ain Boucherit, with most of the evidence being concentrated at AB-Lw level and scarce at AB-Up assemblage (Table 9 and Fig. 9).

**Table 9:** Specimens with anthropic modifications from AB-Lw and AB-Up. Skeletal and taxonomical information, type of damage (cut marks and percussion marks), schematic description, and butchery activity identified have been included. All specimens belong to adult individuals, except those marked with (*). Lat. = laterality; (A) antilopinae; (B) bovidae; (E) equidae

Level	Taxon	Element	Zone	Lat	Percussion marks	Cut marks (number, distribution, delineation, and location)	Butchery activity
AB-Lw	Very large	Rib	Diaphysis	-		2 grouped and oblique slicing marks on ventral side	Evisceration
	Large (E)	M^1/2^	Complete	Left		2 grouped and transversal slicing marks on buccal side	Defleshing
	Large	Rib	Diaphysis	-		2 grouped and oblique slicing marks on ventral side	Evisceration
	Large	Rib	Diaphysis	-		1 isolated and oblique slicing marks on dorsal side	Defleshing
	Large	Rib	Diaphysis	-		2 grouped and oblique slicing marks on dorsal side	Defleshing
	Large (E)	Calcaneus	Complete	Right		1 isolated and oblique slicing marks on lateral side	Skinning
	Medium (B)	Humerus	Diaph + distal end	Right		3 grouped and oblique slicing marks medial side	Defleshing
	Medium (B)	Femur	Diaphysis	-	Percussion pits		Marrow exploitation
	Medium	Long bone	Diaphysis	-		1 isolated and oblique slicing marks on diaphysis	Defleshing
	Medium	Long bone	Diaphysis	-	Cortical conchoidal scar		Marrow exploitation
	Small (B)	Mandible	Symphysis + body	Left		2 grouped and oblique slicing marks on buccal side	Defleshing
	Small	Rib	Diaphysis	-		1 isolated and oblique slicing marks on dorsal side	Defleshing
	Small (B)	Humerus	Metaph + distal end	Left		1 isolated and oblique slicing marks on lateral side	Defleshing
	Small (B)	Radius	Diaph + distal end	Left		2 grouped and oblique slicing marks on lateral side of distal diaphysis	Defleshing
	Small (A)	Metacarpal	Diaph + proximal end	Right		5 grouped and oblique slicing marks on medial side	Skinning
	Small (B)	Metacarpal (*)	Diaph + proximal end	-		2 grouped and oblique slicing marks on anterior side	Skinning
	Small (B)	Tibia	Diaphysis	-		1 isolated and logitudinal slicing marks on anterior mid-diaphysis	Defleshing
	Small (B)	Tibia	Diaphysis	-		1 isolated and oblique slicing marks on anterior mid-diaphysis	Defleshing
	Very small (B)	Radius(*)	Complete	Right		2 grouped and oblique slicing marks on posterior side	Defleshing
	indet	Long bone	Diaphysis	-		1 isolated and transversal slicing marks on diaphysis	Defleshing
	indet	Long bone	Diaphysis	-	Impact		Marrow exploitation
	indet	Long bone	Diaphysis	-	Bone flake		Marrow exploitation
AB-Up	Large (E)	Tibia	Diaph + distal end	Right		3 grouped and transversal slicing marks and scraping marks grouped and longitudinal on anterior and posterior sides	Defleshing; Periostium extraction
	Large (E)	Tibia	Diaphysis	Left	Impact		Marrow exploitation
	Large (E)	Tibia	Diaphysis	Left	Cortical conchoidal scar		Marrow exploitation
	Large (E)	Tibia	Diaphysis	Right		1 isolated and longitudinal slicing marks on lateral side close to tibial crest	Defleshing
	Medium	Femur	Diaphysis	-	Medular conchoidal scar		Marrow exploitation
	Medium	Long bone	Diaphysis	-		1 isolated and oblique slicing marks	Defleshing
	Medium	Long bone	Diaphysis	-	Percussion pits		Marrow exploitation
	Medium	Long bone	Diaphysis	-	Bone flake		Marrow exploitation
	Medium	Long bone	Diaphysis	-	Medular conchoidal scar		Marrow exploitation
	Medium (B)	Long bone	Diaphysis	Right		1 isolated and oblique slicing marks on lateral side	Defleshing
	Small	Tibia	Diaphysis	-	Cortical conchoidal scar		Marrow exploitation

At AB-Up, there are 4 cutmarked bones (0.70%) and 7 percussed bones (1.22%). The cut marks appear on two Equidae tibiae and two medium-sized long bones. They consist of incisions that generally appear isolated, except in one Equidae tibia where they appear concentrated. This tibia also presents scraping marks. Although the bone surfaces of this archaeological level seem to be strongly altered, it is sometimes possible to discern the main characteristics of the cut marks, such as the linear trajectory with V-shape section and internal microstriation or the presence of hertzian cones. The length of the incisions ranges from 5 to 25 mm. The incisions are related in all cases to defleshing activities. The scraping mark could also be associated with the periosteum removal.

Percussion marks (Table 9) have been identified on bones of large-, medium-, and small-sized animals. In the large-sized category, percussion marks include an impact and a cortical scar on two tibiae of Equidae (Fig. 9). In the medium-sized category, one femur and one long bone present medullary scars, another long bone shows percussion pits, and one bone flake has been recorded. Finally, in the small-sized animals, a tibia shows cortical scars.

As for the AB-Lw level, a total of 32 cut marks are recognized on 18 fossils of all animal size categories (Table 9). They are slicing marks with oblique disposition mainly isolated or in groups of two marks and present linear trajectory with V-shape section and internal microstriation (Fig. 9). Two slicing marks are located on the ventral face of a rib fragment of a very large animal, suggesting that it was eviscerated. Eight marks are present on bones of large animals including three ribs, one molar and one calcaneus denoting skinning, evisceration, and defleshing activities. Four cutmarks related to a defleshing activity belong to medium-sized animals, including a humerus and a long bone. The higher number of cutmarked bones (*n* = 8) pertains to small animals (mandible, rib, humerus, radius, two metacarpals, and two tibiae). This is the only size category involving cuts (*n* = 15) in all skeletal parts, suggesting defleshing activities of cranial, axial, and appendicular skeletons, and skinning of lower limbs. There are also three indeterminate cutmarked bone fragments.

The evidence of percussion marks at AB-Lw assemblage is less exhaustive and includes 4 hammerstone percussed bones: percussion pits in a medium-sized femur and a long bone with cortical scars. In addition, there are impacts on an indeterminate bone fragment and a bone flake.

#### Carnivore activity

Evidence of carnivore toothmarks (pits and scores) is documented in both Ain Boucherit archaeological levels. At AB-Lw level, they are recognized on 6 fossil bones (1.81%) (Fig. 7) including a vertebra, 2 humeri and a metacarpal of small-sized animals, a cervical vertebra of a medium-sized animal, and an indeterminate bone fragment. Pits (mean length: 4.54 mm; mean breadth: 4.14 mm) are identified on the cancellous tissues of the two vertebrae. All the scores (mean length: 3.27 mm; mean breadth: 0.65 mm) appear on cortical tissues. Only the cervical vertebra presents evidence of furrowing. Because tooth marks are scant in AB-Lw assemblage, there is not enough data to perform detailed metric and statistical analyses.

**Fig. 10:**
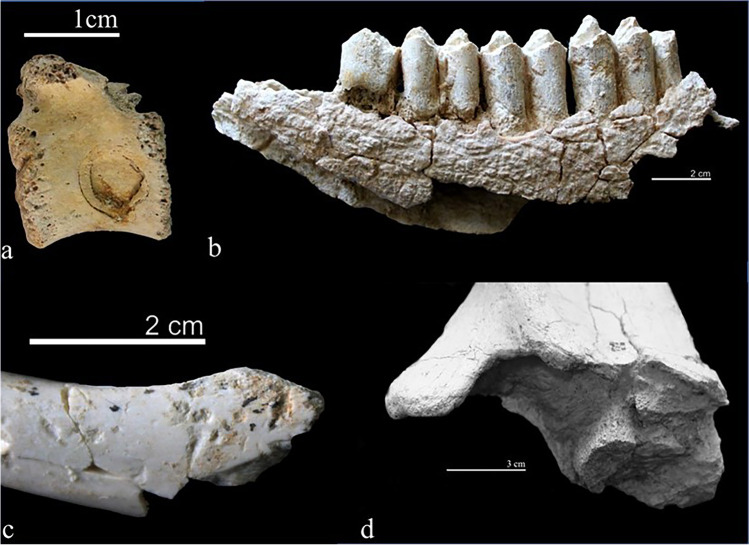
Bone surface modifications from AB-Lw and AB-Up; **a** toothmarks on a small-sized vertebra from AB-Lw; **b** medium-sized bovid mandible from AB-Up with poor preservation of bone surface related to water and plant activity; **c** toothmarks on a small animal tibia fragment from AB-Up level; **d** furrowing on a hippopotamus scapula from AB-Up

However, the carnivore activity at the AB-Up level is more frequent, reaching 9.58% of the damaged fossil remains (Figs. 7 and 10). Whereas the carnivore activity affected almost equally three main animal size categories, they also exploited very large and very small animals (Table 10). The appendicular elements show the greatest number of carnivore damage (78.18%), affecting mainly intermediate limb (20%) and lower limb (14.55%) bones. Cranial and axial elements show lower incidence of carnivore damage, ranging between 5.45 and 7.27%.

**Table 10:** Carnivore damage on skeletal elements from AB-Up level according to animal size category

Skeletal segment	Element	Very large	Large	Medium	Small	Very small	Indet	Total
Skull (3–5.45%)	Skull				1			1
	Mandible		1		1			2
Axial (4–7.27%)	Rib		1	1				2
	Scapula	1						1
	Coxal		1					1
Upper limbs (4–7.27%)	Humerus		1	1	1			3
	Femur		1					1
Intermediate limbs (11–20%)	Radius		2					2
	Ulna				1			1
	Tibia		5	1	1	1		8
Lower limbs (8–14.55%)	Metapodial		2		4			6
	Phalanx				2			2
Indeterminate limbs (20–36.36%)	Long bone		2	8	8	2		20
Indet (5–9.09%)	Flat bone	1		2				3
	Indet			1			1	2
Total		2	16	14	19	3	1	55

Percentage refers to the total remains per skeletal segment with carnivore activity. Indet. = indeterminate fragments.

Most of the damages produced by carnivores at AB-Up are pits (*n* = 27–49.09%) and scores (*n* = 25– 45.45%), but other types of damage also occur, including furrowing (*n* = 8–14.55%), scooping out (*n* = 6–10.91%), modifications produced by digestion (*n* = 6–10.91%) and pitting (*n* = 2–3.63%). Furrowing and scooping out affected mainly large animal bones, while digestion and pitting are observed on small- and medium-sized carcasses (Table 11).

**Table 11:** Frequency of carnivore damage according to size category at AB-Up level. Percentage according to the total damaged bones between brackets

	Pits	Scores	Furrowing	Scooping out	Pitting	Digestion
Very large size	1 (1.82)	1 (1.82)	1 (1.82)	-	-	-
Large size	8 (14.55)	8 (14.55)	4 (7.27)	5 (9.09)	-	-
Medium size	6 (10.91)	7 (12.73)	-	-	1 (1.82)	2 (3.64)
Small size	9 (16.36)	6 (10.91)	3 (5.45)	1 (1.82)	1 (1.82)	4 (7.27)
Very small size	2 (3.64)	2 (3.64)	-	-	-	-
Indet	1 (1.82)	1 (1.82)	-	-	-	-
Total	27 (49.09)	25 (45.45)	8 (14.55)	6 (10.91)	2 (3.64)	6 (10.91)

**Fig. 11:**
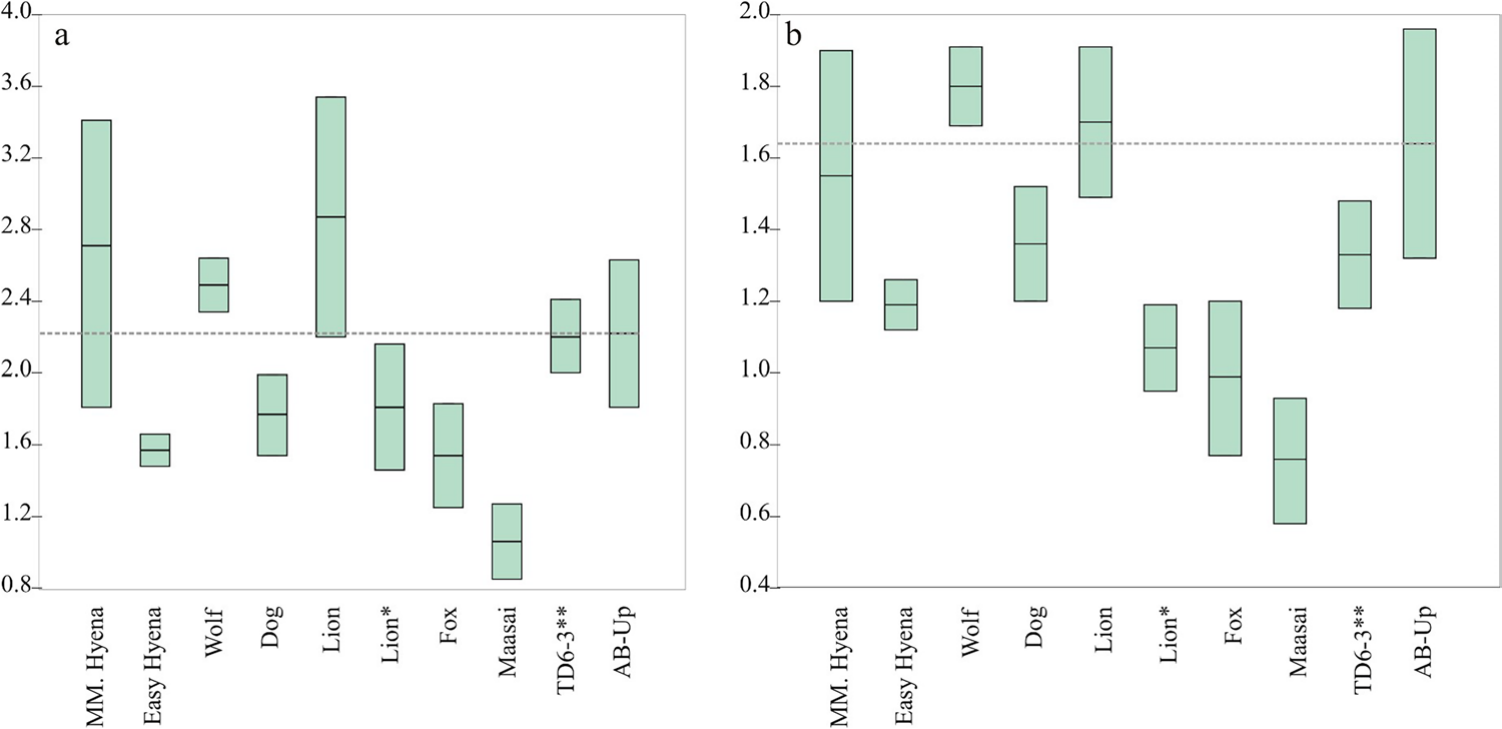
Length (**a**) and breadth (**b**) of pits on shaft documented on fossils from AB-Up compared with experimental data provided by Andrés et al. (2012) and Delaney-Rivera et al. (2009) marked with * and archaeological data provided by Saladié et al. (2019) marked with **

**Fig. 12:**
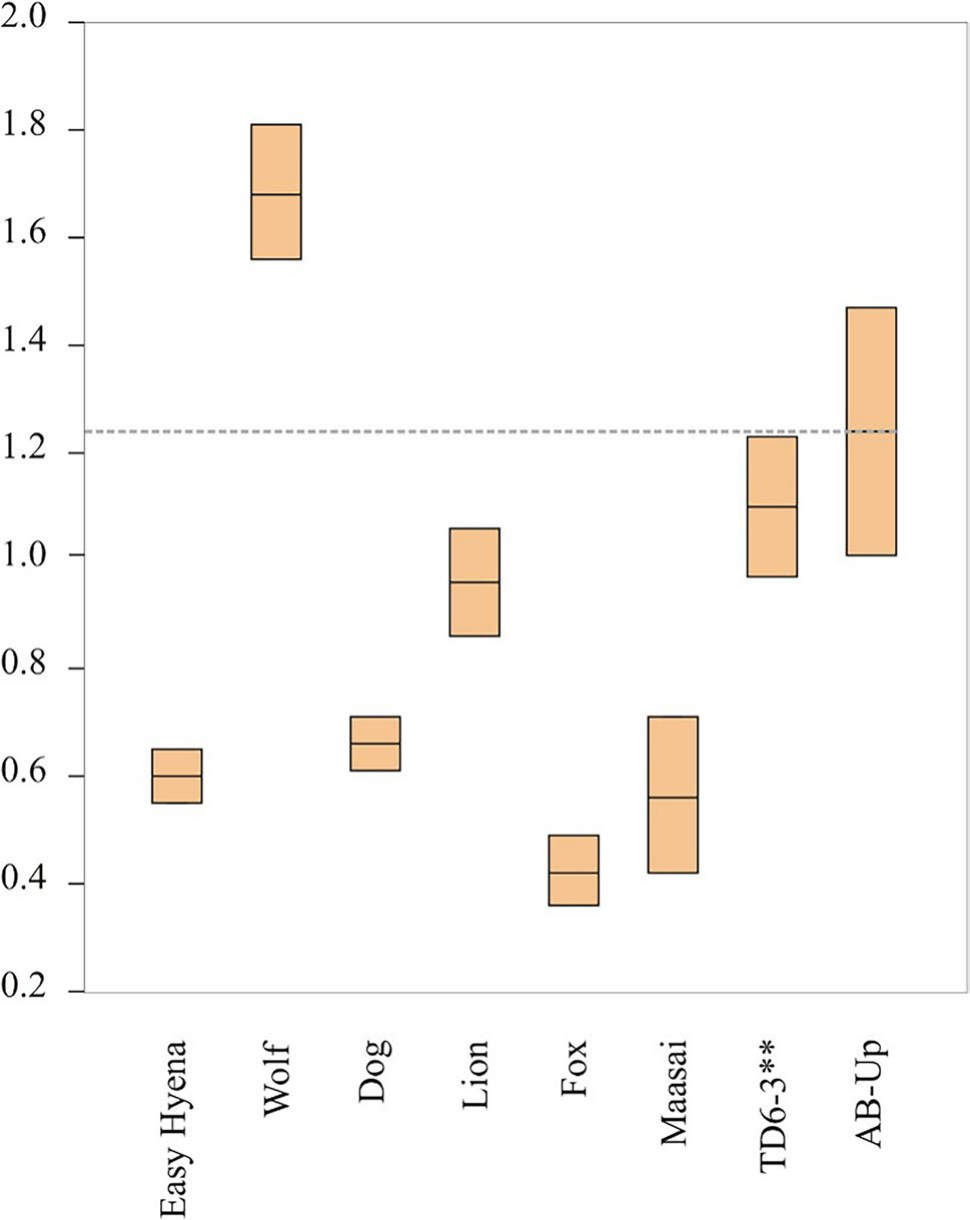
Breadth of scores on shaft documented on fossils from AB-Up compared with experimental data provided by Andrés et al. (2012) and archaeological data provided by Saladié et al. (2019) marked with **

Pits appear primarily in cortical tissue (*n* = 23), with only four pits on cancellous tissue, which, in most cases, could not be measured. For those that it was possible to measure, their dimensions are 2.40 and 1.96 mm, respectively length and width. A total of 27 pits on cortical tissue were measured, providing a mean length of 2.22 mm and mean width of 1.64 mm. Similarly, the scores are located mostly on diaphysis (*n* = 22), with few scores on cancellous tissue having a mean width of 2.32 mm. The 30 cortical scores recorded provide a mean width of 1.24 mm. Comparison of the data with results of either experimental or archeological carnivore damage studies (Figs. 11 and 12) suggests that both AB-Up pits and scores were produced by a large carnivore, such as a hyena, with dimension lightly above those characterizing the Gran Dolina TD6-3 faunal assemblage (Atapuerca, Spain) (Saladié et al. 2019).

**Fig. 13:**
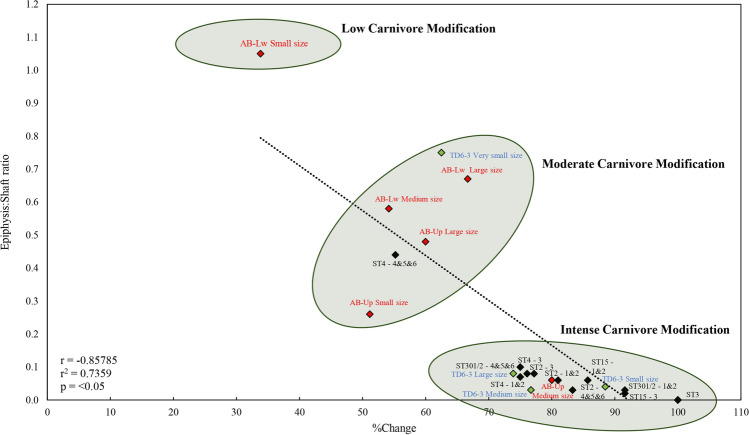
Relation %change and epiphysis-diaphysis ratio of long bones from AB-Lw and AB-Up according to the animal size categories compared with data from ST Olduvai sites (black) provided by Domínguez-Rodrigo et al. (2002) and from TD6-3 (green) provided by Saladié et al. (2019)

The scarcity of tooth marks on epiphysis might be due to a bias in the representation of these skeletal parts caused by carnivore activities, as suggested by the data of furrowing and scooping out damage, at least for the large- and medium-size categories of animals. In this sense, the epiphysis:diaphysis ratio and the percentage of change for long bones (Table 12 and Fig. 13), calculated for the three main size categories, suggest a moderate or low ravaging rate for most animal sizes at Ain Boucherit, except for the medium AB-Up-sized animals where ravaging was high.

**Table 12:** Epiphysis:diaphysis ratio and %change in long bones by size category from AB-Lw and AB-Up levels

		Epiphysis:diaphysis ratio	%Change
AB-Lw	Large size	0.67	66.67
	Medium size	0.58	54.17
	Small size	1.05	33.82
AB-Up	Large size	0.48	60
	Medium size	0.06	80
	Small size	0.26	51.16

## Discussion

Ain Boucherit is an exceptional open-air site with two archaeological levels, AB-Lw and AB-Up, that yielded Oldowan stone tools associated with fossil bones bearing traces of early hominin butchery activities. The assemblages are dated to 2.4 Ma for AB-Lw and to 1.9 Ma for AB-Up, thus documenting the oldest evidence of human occupation in North Africa (Parès et al. 2014; Sahnouni et al. 2018; Duval et al. 2021). The cut marks and percussion marks identified in both assemblages suggest that hominins used lithic tools to exploit meat and marrow of different animal sizes. The exploitation strategies of these resources are different in each level, or, at least, they have given rise to an uneven number of evidence. In both assemblages, there is predominance of bovids over other taxa, with small-sized bovids being the most abundant, followed by medium-sized ones. Skeletally, small- and medium-sized animals are also the best represented (Tables 3, 4, and 5). The presence of large and very large animals is scarce, with Equidae remains dominating in the AB-Up level. The predominance of small- and medium-sized animals is common in Oldowan assemblages, as attested by data from sites such as Gona (Domínguez-Rodrigo et al. 2005; Cáceres et al. 2017a), Kanjera South (Ferraro et al. 2013), FwJj20 (Braun et al. 2010), and several sites from Bed I of Olduvai as FLK Zinj, DS, or PTK (Domínguez-Rodrigo et al. 2021; Cobo-Sánchez 2020; Organista et al. 2023).

Overall, in both assemblages, the skeletal profiles denote a low representation of axial elements and predominance of limb bones, specifically lower limbs. It should be noted that, at AB-Lw, the cranial skeleton of large-sizes animals is not represented. The scarcity of specifically identified teeth has not allowed us to estimate mortality profiles for either assemblage. However, based on the degree of bone epiphyseal fusion, adult individuals predominate.

The anatomical profiles present at Ain Boucherit levels do not seem to be linked to differential preservation of mineral bone density of the remains, since in both levels and in all sizes, there is a null or weak correlation between %MAU and BMD (Table 6). This correlation is statistically significant for the small-size animals in both levels and for the medium-sized animals in AB-Up, being the best sizes represented at the assemblages. Thus, the Ain Boucherit skeletal representation could have involved several taphonomic agents (Fig. 7), including biological agents (hominins and carnivores) or some post-depositional processes. The skeletal profiles based on the food utility indexes suggest that the nutritional strategy at AB-Lw likely corresponds to a strategy close to the complete transport of both, medium- and small-size animals (Table 7). At AB-Up, the strategy is similar for small carcasses, but nutrients are less represented for medium and large animals, pointing toward an unconstrained strategy.

Post-depositional modifications identified include those characteristics of open-air environments, such as weathering, water abrasion, and chemical corrosions linked to plant activity. The exfoliation and loss tissue on bones detected at AB-Up, related to extreme stages of weathering (Behrensmeyer 1978), suggest a longer exposure to subaerial damage than AB-Lw. Water abrasion, attested by the occurrence of rounded and polished bones, suggests that the bones were dry or weathered before the beginning of surface modifications. The Ain Boucherit sediments are predominantly fine-grained particles, typical of a floodplain setting that is known to favor the development of abrasion resulting in more rounded than polished surfaces, as suggested by experimental studies (Fernández-Jalvo and Andrews 2003). The highest incidence of this type of alteration at AB-Up suggests that water flows were more persistent than at AB-Lw. Water flows at Ain Boucherit should have been of low or moderate energy since heavily abraded bones are rare. There is no evidence of fluvial transport in either archaeological level (Fig. 8), suggesting a minimal hydric disturbance of the faunal assemblages. The null or weak correlation between %MAU and bone mineral density supports the low incidence of water currents on the anatomical composition of both assemblages. The incidence of these agents and processes is more visible at AB-Up than at AB-Lw, resulting in poor preservation of bone surfaces in the former, although this has not been an impediment to analyze the surface of most of the specimens.

The scarcity of complete bones is the common feature in both assemblages. Most of the excavated bone fragments do not exceed 5 cm in length. The data suggest that the breakage of the bones occurred mostly in green state, since curved fractures with oblique angles and smooth surfaces predominate (Villa and Mahieu 1991). When comparing bone surface planes with experimental assemblages (Alcántara-García et al. 2006; Coil et al. 2017; Moclán et al. 2019), small- and medium-sized animals show values close to anthropogenic bone breakage models, while large animals from AB-Up provide values related them to carnivore action. The bone breakage pattern, together with surface modifications, points out to the involvement of hominins and carnivores in accessing animal carcasses of different sizes but unevenly. Early hominins were more involved in accumulating the AB-Lw faunal assemblage than at the AB-Up, while carnivores were more active at AB-Up (Fig. 7 and Table 9).

At the AB-Lw level, early hominins accessed all animal carcass sizes and all anatomical parts, although it is more preponderant in small- and medium-sized animal carcasses, especially limb bones (Table 9). The location of the cut marks indicates that early North-African inhabitants were involved in skin removal, evisceration, and defleshing of animal carcasses (Nilssen 2000). This evidence is not consistent with usual secondary access to carcasses abandoned by felids, since these predators would not have left meat packages usable in small sizes (Domínguez-Rodrigo et al. 2021), which are the animals with the highest hominin activity at AB-Lw. Furthermore, the predominance of cut marks on the intermediate and upper limbs supports, at least, early access to small- and medium-sized carcasses. (Bunn 2001; Domínguez-Rodrigo and Pickering 2003; Pickering and Domínguez-Rodrigo 2006; Pickering and Egeland 2009). These patterns are consistent with those documented in other Oldowan faunal assemblages in East Africa such as FwJj20 (Braun et al 2010), FLK Zinj, DS, PTK (Domínguez-Rodrigo et al. 2007a, b; Domínguez-Rodrigo and Pickering 2017; Bunn and Pickering 2010; Parkinson 2018; Oliver et al. 2019; Cobo-Sánchez 2020; Domínguez-Rodrigo et al. 2021; Organista et al. 2023), and Kanjera South (Ferraro et al. 2013), where studies even concluded to regular access of small-sized carcasses by early hominins. The regular hominin access to small and medium sizes could have also occurred at AB-Lw. In addition, the cut marks located on the ventral surface of ribs of larger-sized carcasses are unequivocally related to viscera extraction (Nilssen 2000). We can, therefore, propose that they had also early access to large animal carcasses, at least sporadically. We do not have enough data to conclude on the kind of strategy for meat acquisition (e.g., hunting or confrontational scavenging), but, so far, the available evidence has strongly shown that they had primary and early access to animal tissues. The scarcity of carnivore damage at AB-Lw (1.81%), together with the abundance of preserved epiphyses (Table 12), indicates a low carnivore ravaging in this archaeological level (Fig. 13), which is suggestive of low competition with carnivores for resources favoring primary access by early hominins.

For the AB-Up faunal assemblage, the dynamics seem to be different. Carnivore involvement is greater, reaching almost 10% of modified bone surfaces and affecting all animal size categories. This fact—tooth marks are the main source of damage, although epiphysis consumption (furrowing and scooping out), pitting, and bone digestion have also occurred. Considering the dimensions of the tooth marks (Figs. 11 and 12) and their comparison with published data (Andrés et al. 2012; Delaney-Rivera et al. 2009), carnivore damage to AB-Up bones could have been produced by large carnivores such as hyenas (Saladié et al. 2019). In addition to toothmark size, and as criteria for identifying hyena activity should not be taken individually (Kuhn et al. 2010), the evidence of furrowing and scooping out recognized on very-large-sized animal bones might suggest their involvement as bone accumulators at the AB-Up level (Fig. 10), and possibly breaking bones, regarding the longitudinal/oblique ratio provided (Table 8). Yet, we would also expect a greater number of tooth marks (Blumenschine 1988), as well as more digested bones and presence of coprolites (Pickering 2002; Marra et al. 2004; Egeland et al. 2008; Saladié et al. 2019). Digestion effects are seen on only 1.06% of fossil bones of AB-Up, and no coprolites were found in this level. Therefore, it does not appear that hyenas were the main bone-accumulating agent, and we must consider that their activity occurred secondarily. The ratio epiphysis:diaphysis and the percentage of change in all animal size categories suggest that the carnivore involvement is generally low or moderate, except for the medium-sized animals of AB-Up, where there is a significant bone ravaging by carnivores (Fig. 13). The AB-Up values for medium-sized animals are consistent with values from ST Olduvai sites (Domínguez-Rodrigo et al. 2002) and from TD6-3 (Saladié et al. 2019), where carnivore bone damage is high. Thereby, we suggest that large carnivores were involved in the bone accumulation, but they were not the only agents and, perhaps, not always the first.

Hominin tool–induced damage at AB-Up is mainly present in the medium- and large-sized animals. There is only one bone of a small-sized element that shows marks produced by humans, recalling patterns from the nearby El-Kherba Oldowan site, where consumption of large animals was more abundant than of small ones (Sahnouni et al. 2013). At AB-Up, the evidence is mostly related to marrow procurement, and only four bones bear cutmarks. These cutmarked bones indicate defleshing of medium-sized long bones and Equidae tibias and subsequent breakage (presence of scraping marks). The large- and medium-sized percussed bones for marrow acquisition could suggest, for the case of AB-Up faunal assemblage, hominins were more interested in the marrow than in the meat. This could be in line with the hypothesis that hominin predatory behavior was initiated through secondary access to carcasses abandoned by carnivores, focusing on marrow acquisition rather than on consuming meat (Thompson et al. 2019). The AB-Up lithic assemblage comprises 47.8% cores or core forms (Table 1), suggesting the occurrence of an important percussive activity by hominins. Yet, there is also 25% of flakes that suggest cutting activities. At this point, it is important to recall that AB-Up bone surfaces are poorly preserved, which could have prevented recognizing cutmarks, since there is a relationship between the readable bone surfaces and the low frequency of cutmark identification according to Pobiner et al. (2008). Therefore, we cannot assert that AB-Up hominins consumed marrow at the expense of meat. Rather, it is an issue of obliteration of traces due to the involvement of multiple post-depositional agents in an open-air context.

At AB-Up, the dynamics developed by hominins and carnivores is more complex than at AB-Lw. In the former, both predators had to share space and compete for animal resources. While hominins focused mainly on large- and medium-sized animals, carnivores exploited all sized animals, including ravaging those carcasses discarded by hominins. The absence of overlapping marks does not allow us to discern exactly their respective order of access to animal carcasses. Therefore, both agents acquired animal carcasses and consumed their meat and marrow, contributing to the formation of the assemblage.

Hominin animal exploitation at Ain Boucherit is near contemporary with the oldest evidence of butchery activities at Gona in East Africa (Semaw et al. 2003; Domínguez-Rodrigo et al. 2005; de Heinzelin et al. 1999; Cáceres et al. 2017a). Yet, it is more similar to those reported from younger sites dated around 2.0 Ma from that eastern area, where regular consumption of animal resources is attested (Bunn et al. 1980; Potts 1988; Kimbel et al. 1996; Domínguez-Rodrigo et al. 2007a, b; Braun et al. 2010; Ferraro 2007; Ferraro et al. 2013; Oliver et al. 2019; Cobo-Sánchez 2020; Domínguez-Rodrigo et al. 2021; Organista et al. 2023). The fact that when hominin activity increases (AB-Lw), carnivore activity is lower and vice versa (AB-Up) has also been documented at the Bed I sites of Olduvai (Domínguez-Rodrigo et al. 2007a, b; Cobo-Sánchez 2020). This suggests that, in chronologies prior to 2 Ma, hominins were already able to successfully obtain meat resources without relying on the remains abandoned by carnivores. Ain Boucherit sites yield the first evidence of manufacturing and using stone tools in meat cutting and marrow acquisition in North Africa. This evidence suggests that regular access to meat resources, with all the underlying behavioral implications (group organization, food sharing, etc.), does not seem to be an isolated behavior that occurred in some places in East Africa but should be understood as a generalized behavior of hominids that started early.

## Conclusions

Ain Boucherit currently documents the earliest archaeological traces of human occupation in North Africa. By 2.4 Ma, early hominins already had the ability to manufacture Oldowan stone tools and use them to consume meat and marrow from carcasses of different animal sizes. At the older Ain Boucherit level (AB-Lw), we can conclude that hominin groups had early access to small-sized bovids regularly, which could have also extended to medium-sized carcasses and, at least, sporadically to large animals. In the younger Ain Boucherit level (AB-Up) dated at 1.9 Ma, although primary access to carcasses by hominin is documented, there must have been a great competition between hominins and carnivores for meat acquisition. This evidence is similar to that provided by younger sites in East Africa and thus extends the chronology of regular hominin access to meat resources, suggesting that human groups may have developed early on the complex and collaborative behavior.

The Oued Boucherit region had the necessary resources (biotic and abiotic) to the development of these early occupations in North Africa and to broaden in time as attested by the younger sites of El-Kherba (Oldowan, 1.8 Ma) and Ain Hanech (Acheulean, 1.7 Ma). Thus, future studies planned at Ain Boucherit will allow to deepen the knowledge of the subsistence strategies and behavior carried out by the first inhabitants of North Africa.

## Data Availability

Not applicable.
